# Effects of Nozzle Temperature on Mechanical Properties of Polylactic Acid Specimens Fabricated by Fused Deposition Modeling

**DOI:** 10.3390/polym16131867

**Published:** 2024-06-29

**Authors:** Fernando Rivera-López, María M. Laz Pavón, Eduardo Cabello Correa, María Hernández Molina

**Affiliations:** Departamento de Ingeniería Industrial, Escuela Superior de Ingeniería y Tecnología, Universidad de La Laguna, Apdo. 456, E-38200 San Cristóbal de La Laguna, Santa Cruz de Tenerife, Spain

**Keywords:** polylactic acid, 3D printing, fused deposition modeling, nozzle temperature, mechanical properties

## Abstract

This paper investigates the effect of nozzle temperature, from 180 to 260 °C, on properties of polylactic acid (PLA) samples manufactured by fused deposition modeling (FDM) technology. The main objective of this research is to determinate an optimum nozzle temperature relative to tensile, flexural and compressive properties of printed specimens. After manufacturing, the samples exhibit an amorphous structure, without crystallization effects, independently of the fabrication temperature. In order to determine the influence of printing temperature on mechanical properties, uniaxial tensile, three-point flexural and compression strength tests were carried out. The obtained results suggest that a relative low printing temperature could reduce the material flow and decrease the density of the final prototype, with a negative effect on both the quality and the mechanical properties of the pieces. If temperature increases up to 260 °C, an excess of material can be deposited, but with no significant negative effect on mechanical parameters. There is an optimum nozzle temperature interval, depending on the considered piece and test, for which mechanical values can be optimized. Taking into account all tests, a recommended extruder temperature interval may be identified as 220–240 °C. This range encompasses all mechanical parameters, avoiding the highest temperature where an excess of material was observed. For this printing temperature interval, no significant mechanical variations were appreciated, which corresponds to a stable behavior of the manufactured specimens.

## 1. Introduction

Three-dimensional (3D) printing is considered a promising additive manufacturing (AM) technology for the fabrication of customized prototypes [1,2,3,4,5]. The process consists of joining material layer by layer upon each other with the aim of obtaining the desired object. Among all the AM methods, fused deposition modeling (FDM) [6,7,8,9] has experienced an incredible growth due to the interest of commercial users and professionals, low cost, flexibility in geometry, and simplicity. With respect to possible thermoplastic materials, polylactic acid (PLA) is not an expensive printing material, widely employed in important sectors, such as aeronautical [10], automotive [11] and education [12]. PLA is an eco-friendly thermoplastic polymer that can be obtained from renewable resources such as, among others, sugarcane, corn, wheat, cassava, barley starch or cellulose [13,14,15] and it can be considered as compostable under specific conditions [16].

When PLA is employed as raw material for FDM, it is very important to study the influence of manufacturing parameters on mechanical properties for an optimal selection of the printing settings. As can be seen in the literature, many researchers are investigating the achievement of better mechanical properties through parameter optimization. Lanzotti et al. [17] reported the impact of the layer thickness, the infill orientation and the number of shell perimeters on mechanical properties of pieces. A decrease in strength was observed as the infill orientation approached 90°, and an increase as the perimeters increase. The strain value reaches its maximum at 0.15 mm of layer thickness. Rajpurohit and Dave [18] presented a tensile properties comparison between unidirectional and bidirectional raster angle for different layer height and raster width. For unidirectional raster angle, the highest tensile strength was obtained at 0° raster angle, whereas for bidirectional raster this was 45°. Vălean et al. [19] evaluated the influence of building orientation and different thickness on main tensile properties. In that work, it was observed that the spatial printing orientation has less influence on the Young modulus and more influence on the tensile strength, and the increase in the number of layers decreases the value of both parameters. Corapi et al. [20] investigated the correlation of the basic tensile proprieties of materials with three perpendicular growing directions. The results pointed out that the highest elastic modulus and the ultimate tensile strength correspond to the horizontal specimens. Afrose et al. [21] studied the effect of the build orientation on fatigue behavior of specimen parts. PLA samples manufactured in x-direction reach the highest tensile stress, in the range of 60–64%, of that of molded raw material. In Lui et al. [22], the tensile strength, flexural strength and impact strength were studied, considering the deposition orientation, the layer thickness, the deposition style, the raster width and the raster gap, presenting a set of optimal process parameter combinations to optimize the mechanical properties of FDM specimens. Christiyan et al. [23] considered different orientations, layer thicknesses and printing speeds for the determination of flexural strength. The best results were obtained for 0° orientation due to the good bonding between layers. Sajjad et al. [24] investigated the effect of multiple infill on the mechanical properties of FDM built specimens, concluding that the combination of rectangular and triangular patterns has optimum strength-to-weight ratio along with minimal production cost.

Among all the printing parameters, the nozzle temperature is considered as one of the most important variables to take into account [25]. The extrusion nozzle melts the thermoplastic filament, the printing temperature being an evident influence on the physical properties of the final piece. For this reason, the motivation of this study is to provide knowledge and advances in both the manufacturing processes parameters and the mechanical properties of the fabricated specimens. Recently, Popescu et al. [26] considered that nozzle and bed temperatures are insufficiently studied in the literature, representing a niche for further studies. Nowadays, there is little available information about the effect of the extrusion temperature on mechanical properties of PLA specimens fabricated by 3D printing FDM. Only a few research papers on board tensile and bending consider the nozzle temperature as a variable [27,28]. Akhoundi et al. [27] studied the effect of nozzle temperature on the tensile strength and the modulus of printed PLA parts, observing that the enhancement of extrusion temperature increases the raster and layer bonds. Song et al. [28] varied the extrusion temperature from 200 to 240 °C, concluding that mechanical properties can be improved by optimizing the temperature due to the minimization of porosity. With respect to other materials, it is worth highlighting the work of Ding et al. [29], where the density of poly-ether-ether-ketone (PEEK) and polyetherimide (PEI) specimens, printed by FDM, improves with the increase in the nozzle temperature because the air pores are partially discharged. In the research of Foppiano et al. [30], 231 °C nozzle temperature for 3D printed acrylonitrile butadiene styrene (ABS) was optimum for highest tensile properties.

In this work, an intense study is carried out on a wide nozzle temperature interval, from 180 to 260 °C, reporting the printing temperature effects on the tensile, bending and compressive mechanical parameters of PLA specimens. This polymer has been chosen because actually PLA is one of the most widely used materials in the world for FDM manufacturing. In addition to the lack of studies with respect to the effect of nozzle temperature on mechanical properties of 3D printed PLA specimens, as mentioned earlier, it is worth pointing out the main novelties of this paper. The range in the studied printing temperature is the widest registered for the study of mechanical properties. This research is also the first that reports, in one paper, on tensile, flexural and compression studies of PLA specimens, varying the nozzle temperature. Finally, the work is of great interest because of the absence of manuscripts reporting the influence of extrusion temperature on compressive properties. Moreover, the consideration of printing temperature is very important because it is related to the manufacturing cost of energy.

## 2. Materials and Methods

### 2.1. Materials and Processing Parameters

As raw material, a sapphire SMARTFIL PLA filament of 1.75 mm diameter (from Smart Materials 3D) was selected. This PLA consists of a mixture of 98% polylactide resin (N° case 9051-89-2, with a high molecular weight (>1000) and <1% of additive pigments. It is not expected to be toxic but, if the filament is burned carbon monoxide, carbon dioxide and toxic aldehydes are released. The manufacturer provides a density value of 1.24 g/cm^3^, a recommended printing bed temperature of 60 °C and an extrusion temperature range of 200–240 °C. As moisture absorption could adversely affect PLA filament, and consequently the mechanical properties of printed specimens, after the PLA packages were opened, they were preserved in vacuum, with the aim of minimizing the effect of moisture absorption.

**Figure 1:**
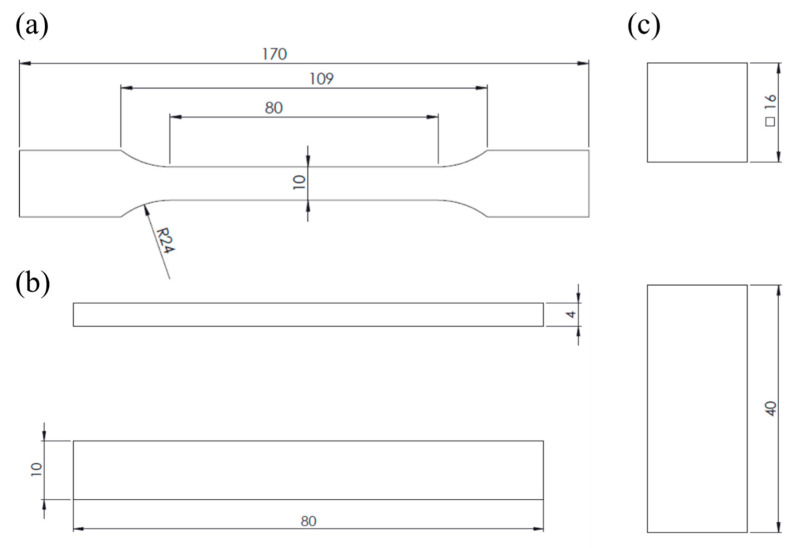
Specimens for (**a**) tensile, (**b**) flexural and (**c**) compressive tests. Dimensions are in mm.

The geometry and dimensions of the samples for this study were selected according to ISO 527-2:2012 [31], ISO 178:2019 [32] and ISO 604:2002 [33] standards for uniaxial tensile, three-point flexural and compression strength tests, respectively. The visual perspectives of the sample types with their main dimensions, according to the standards, are shown in Figure 1. The weights and dimensions of all printed samples can be seen in Tables S1–S4.

The prototypes were designed with SOLIDWORKS 2018 software and saved in surface tessellation language (STL) format. STL files were exported to a slicer (PrusaSlicer 2.3, version 2.8.0), where the manufacturing parameters were selected and saved into a G-Code. The samples for mechanical testing were manufactured from G-Codes, by FDM using a commercial desktop Creality CR-10-S4 3D printer. During printing, fixed parameters were layer width of 0.4 mm, layer height of 0.2 mm, 100% infill pattern density, bed temperature of 60 °C and deposition speeds of 40, 50 and 20 mm/s for perimeters, infill and first layer, respectively. The extruder temperature was changed, as the variable parameter in this work. All the printing parameters are summarized in Table 1.

**Figure 2:**
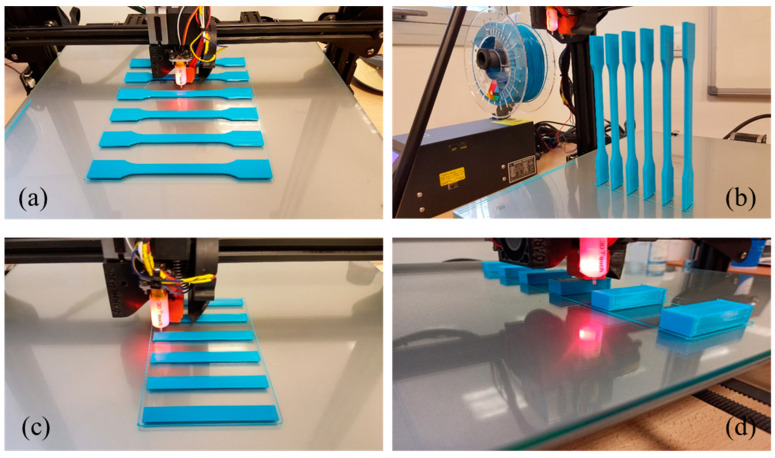
Specimens for tensile tests in (**a**) horizontal and (**b**) transversal printing directions, (**c**) for flexural tests and (**d**) for compression tests.

It is worth noting that, before each printing, the bed was alienated by a coupled automatic calibration system, the 3D Touch sensor. In addition, both temperature and humidity were controlled during manufacturing by a 6 channel MISOL ambient control station (see Table S5). Figure 2 shows printing of different manufactured series. For all tests, a series of six specimens was printed for each temperature. In the case of the tensile samples, two different directions were considered, horizontal (see Figure 2a) and transversal (Figure 2b) orientations.

### 2.2. Thermal Characterization

Thermal characterization of the as-received PLA filament was carried out mainly to know the melting temperature. Differential Scanning Calorimetry (DSC) was performed in a calorimeter (Discovery DSC 025, TA Instruments). Thermogravimetric (TG) and Differential Thermogravimetric (DTG) analysis were performed in a thermal analyzer (Discovery SDT 650, TA Instruments). All the samples were sealed in an aluminum hermetic pan and heated at a rate of 10 °C/min under 50 mL/min of nitrogen gas flow. For DSC measurement, an amount of 7.029 mg of PLA sample was heated from room temperature, 25 °C, to 300 °C. For TG and DTG analysis, an amount of 8.471 mg of the sample was heated from 25 to 500 °C. The analysis of the data was carried out according to ISO 11357-1:2016 standard [34].

### 2.3. X-ray Diffraction (XRD)

The XRD analysis of manufactured specimens was performed in a PANalytical Empyrean diffractometer by using a Cu anode Cu_Kα1,2_ radiation (λ_Kα1_ = 1.5406 Å and λ_Kα2_ = 1.5444 Å) in 0.02626° steps throughout the 5–80 2θ range.

### 2.4. Scanning Electron Microscopy (SEM)

SEM is very useful for investigation of the internal morphology of the printed samples through the fracture cross-sections. The analysis of the fractured samples provides important information that can be related to the mechanical properties. To obtain SEM images, a ZEISS EVO 15 electron microscope, with an acceleration of 15 kV, was used. The samples were prepared cutting small parts near the fractured edge and the surfaces were sputter-coated with a thin layer of gold.

### 2.5. Mechanical Testing

**Figure 3:**
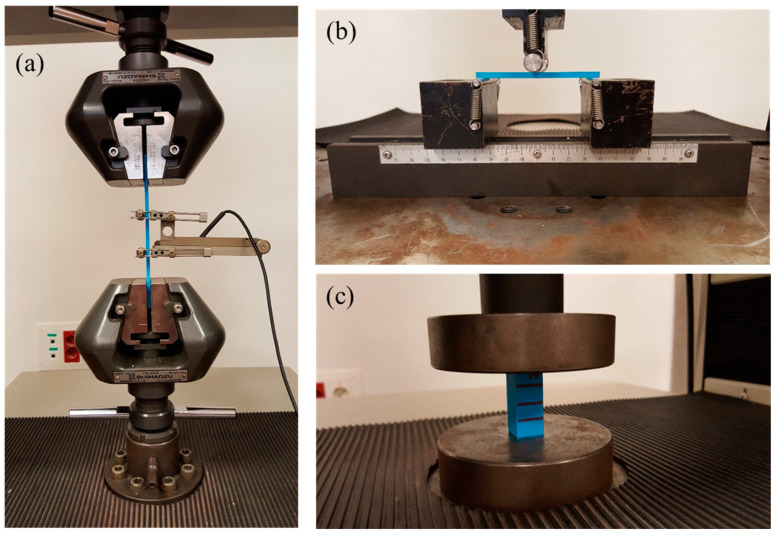
Images of the (**a**) uniaxial tensile, (**b**) three-point bending, and (**c**) compression tests of PLA samples in the electro-mechanical universal testing machine.

The uniaxial tensile, three-point flexural and compression strength tests were carried out in an electro-mechanical universal testing machine (AUTOGRAPH AG-IS, with a load cell capacity of 50 kN) (see Figure 3), according to ISO 527-1:2019 [35], ISO 527-2:2012 [31], ISO 178:2019 [32] and ISO 604:2002 [33] standards, respectively. This machine consists of two crossheads, a fixed down crosshead and a mobile upper, for applying tension to the samples. The mechanical tests were controlled by TRAPEZIUM X software.

For the tensile tests, the specimens were held between grips, with an initial distance of 115 mm (see Figure 3a). The samples were tested, up to failure, at a constant rate of 1 mm/min, which is in accordance with the standard recommendation for this sample geometry. The elongation of the samples during testing was measured, along the direction of the applied force, using an axial 50 mm gauge length extensometer (MFA-25). The experimental registered values from tensile tests were force and elongation and, from these magnitudes, the stress and the strain were obtained for graphical representation and analysis. From the stress–strain curves, the tensile modulus, the tensile strength and the elongation at break were obtained. The tensile modulus was evaluated from the slope through a linear fit in the region from 0.05% to 0.25% of strains, the tensile strength corresponds to the maximum stress value, and the elongation at break is the maximum elongation reached during the test.

Flexural tests were carried out under three-point bending configuration (see Figure 3b), centering the loading nose at the midpoint of the samples. The distance between the support points was 64 mm, calculated from the expression *L* = (16 ± 1)*h*, where *h* is the thickness of the samples (4 mm, see Figure 1b). The crosshead vertical displacement was maintained constant at a rate of 0.5 mm/min. The registered data from bending tests are load and displacement, which were graphically represented as load–displacement. From theses curves, the flexural modulus (*E_f_*), the flexural strength (*σ_f_*) and the flexural strain at break of the outer surface at mid-span (*ε_f_*) were evaluated. The flexural modulus was calculated, according to the ASTM D790-03 standard [36]

(1) $$E_{f}=\frac{L^{3}m}{4bh^{3}}$$

where *L* corresponds to the distance between the support points, *m* is the slope of the load-displacement curve evaluated in the linear region, and *b* and *h* are the width and thickness of the sample, respectively. The flexural strength is defined as in [32,36].

(2) $$\sigma_{f}=\frac{3FL}{2bh^{2}}$$

where *F* is the maximum value of the force. Finally, the flexural strain at break is calculated according to the expression [32,36].

(3) $$\varepsilon_{f}=\frac{6\delta h}{L^{2}}$$

where *δ* is the maximum mid-span deflection.

For compression tests, the specimens were situated between the surfaces of the compression disks, ensuring alignment of its longitudinal axis with the vertical axis of the universal testing machine, the bases of the samples parallel with the surface of the compression tools (see Figure 3c). For determination of compressive properties, all series were tested under axial download compression, with a constant crosshead displacement rate of 1 mm/min, up to 45 kN force, which corresponds, approximately, to a strain of about 60–70%. The experimental values obtained from this test were force and displacement and, from these magnitudes, the stress and the strain were calculated for both graphical representation and subsequent analysis. From the stress–strain curves, the compressive modulus and the compressive yield strength were obtained. The compressive modulus was evaluated from the stress–strain curves, calculating the slope through a linear fit in the elastic region, from 1.5% to 2.5% of strain, and the compressive yield strength is the stress at the yield point. The initial toe part was avoided, because it can be considered as an accommodation of the upper compression disk on the sample surface.

All the parameters are expressed as the mean of five obtained values for each set and the standard deviation. These mean values are evaluated by the use of analysis of variance (ANOVA) in order to determinate correlations when the nozzle temperature changes. The significant correlation is established at a 95% confidence interval level (*p*-value < 0.05).

## 3. Results and Discussion

### 3.1. Thermal and XRD Analysis

**Figure 4:**
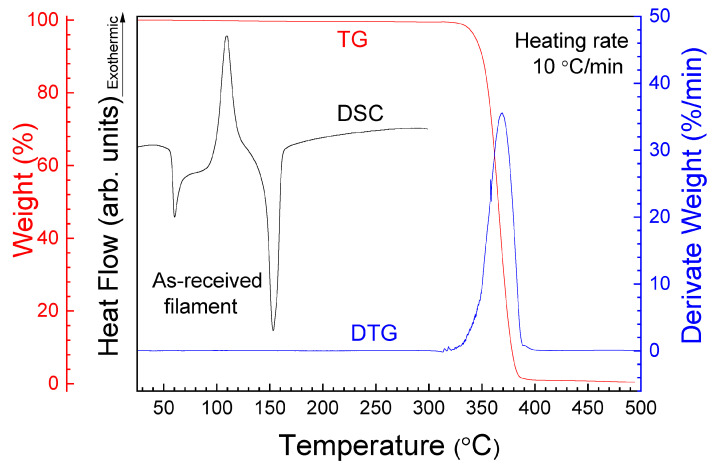
DSC, TG and DTG curves of the as-received PLA filament.

With the aim of studying the thermal stability of PLA filament, DSC, TG and DTG curves of the as-received PLA filament are registered and presented in Figure 4.

According to the TG and DTG graphs, during the first stage, a slight loss of weight can be observed when temperature increases, which could be related to water evaporation. In the second stage, at about 320 °C, the decrease in weight could be associated with the decomposition of the PLA matrix. Around 352.6 °C is the onset temperature of thermal degradation (with 0.51% of mass loss), and 377.6 °C the temperature for complete degradation (with 99.13% of mass loss). The DSC curve shows three peaks. The first endothermic peak is observed at 58 °C, which corresponds to the glass transition temperature (*T_g_*). According to this value, a slightly higher temperature of 60 °C was selected for the bed printing in order to achieve an optimal adhesion of the printed sample to the printing bed [37]. Only one exothermic peak, at around 109.1 °C, is related to the crystallization temperature (*T_c_*), and the last endothermic peak centered at around 153.3 °C can be attributed to the fusion temperature (*T_m_*). The *T_g_* value is in good agreement with that reported by the manufacturer (60 °C) and for the PLA studied by other authors, such as the 61 °C for first heating of the as-received filament reported by Song et al. 2017 [28], or 58.7 °C obtained by Chandran et al. [38]. For *T_c_* and *T_m_*, no comparison is possible with raw manufactured material because these values are not included in the data sheet. The value of *T_m_* was considered as initial reference for printing. According to DSC, TG and DTG results, the interval for printing from 180 to 260 °C can be considered as a safe range for manufacturing temperature. Then, the extruder temperature is changed, for each serial, from 180 to 260 °C, steps of 20 °C. However, it must be noted, according to the results of Figure 4, that high printing temperatures can affect the thermal stability of the printed samples. The effect of temperature on structure is discussed in the next paragraphs and the consequences for mechanical properties in the following sections.

**Figure 5:**
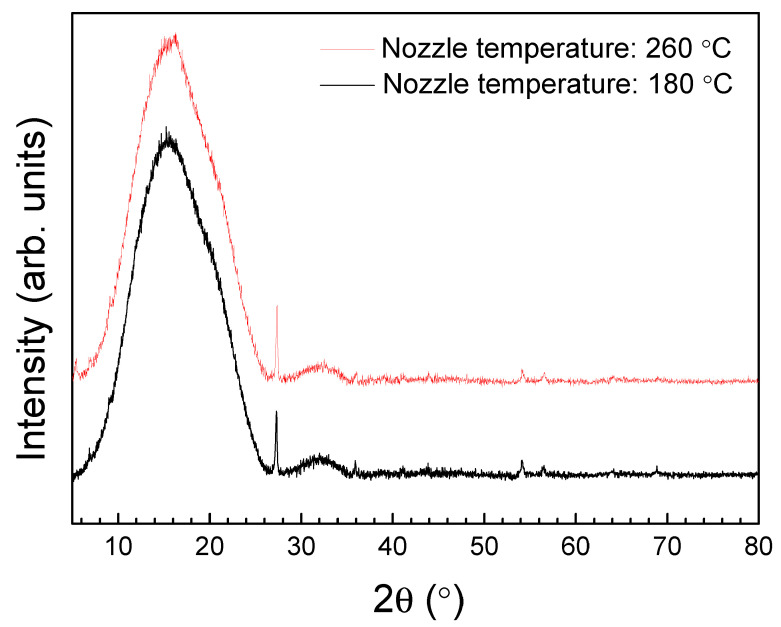
XRD patterns of PLA samples manufactured at 180 and 260 °C.

Once selected, the printing interval, XRD analysis was carried out with the aim of controlling the internal structure of the printed samples. Figure 5 shows the XRD curves of PLA probes after manufacture at 180 and 260 °C.

As can be seen in Figure 5, a similar large broad band, centered at around 16°, is present for both samples, which is typical of amorphous structures without long-range order. Two narrows peaks can also be observed at around 27° and 54°, which indicate a certain crystallinity. The degree of crystallinity, for each sample, was evaluated from the ratio of the integrated area of these narrow peaks and the total integrated area. A percent of 3% of crystallinity was obtained for both printed samples at 180 and 260 °C. Because the percentage of crystallinity for pure PLA (natural without color pigment) should be in the 0–1% range [39], this crystallinity could be associated with the sapphire dye or other additive included in the raw PLA by the manufacturer. For this work, the most important result is that neither crystallization nor structural changes were produced when printing at different temperatures.

### 3.2. Tensile Tests

**Figure 6:**
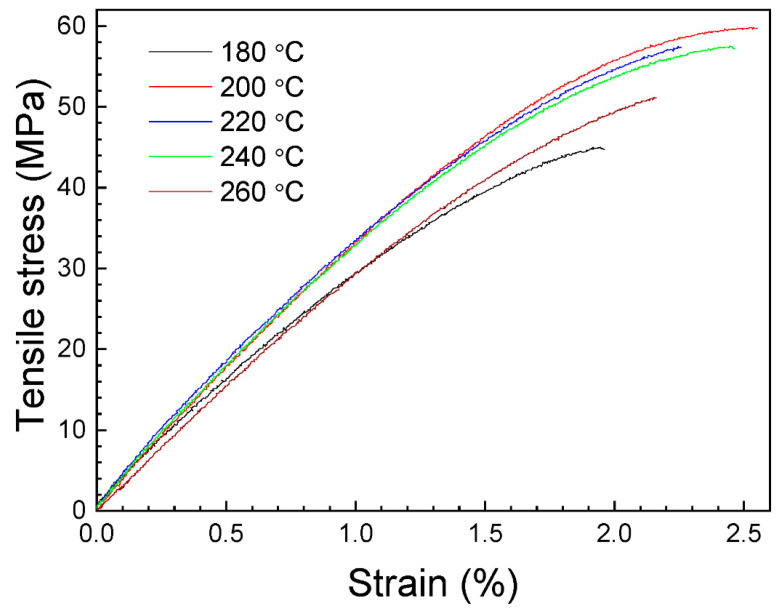
Representative stress–strain curves of the horizontal printed tensile specimens.

Figure 6 shows representative stress–strain curves obtained from the experimental tensile tests of the horizontal manufactured samples. According to Figure 6, initially tested specimens exhibit a quasi-linear behavior in agreement with Hooke’s law. After the first elastic region, the samples present plastic deformation, until reaching the maximum stress value, where failure is produced. It is worth noting that some specimens broke outside of the extensometer region, but all data were considered in this research because, for all the samples, the break is located inside the narrow cross-section of the dog bone.

**Figure 7:**
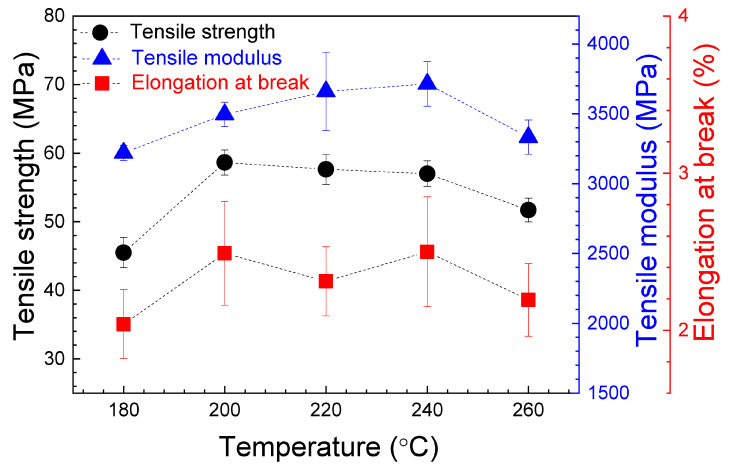
Average values of the tensile strength, the tensile modulus and the elongation at break for the tensile specimens, as a function of nozzle temperature. Data correspond to the horizontal printed samples. The error bars correspond to the standard deviations. The dashed lines are guides for viewing.

Figure 7 shows the relative dependence of the tensile strength, the tensile modulus and the elongation at break on nozzle temperature. The average values of these parameters, with their standard deviation, are presented in Table 2.

**Figure 8:**
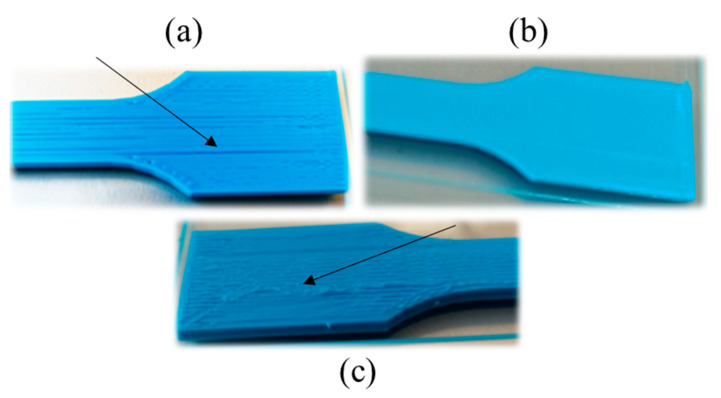
Images of the extreme of tensile specimens under different nozzle temperatures of (**a**) 180 °C, (**b**) 200 °C and (**c**) 260 °C. Arrows indicate the air gap in (**a**) and excess of material in (**c**).

**Figure 9:**
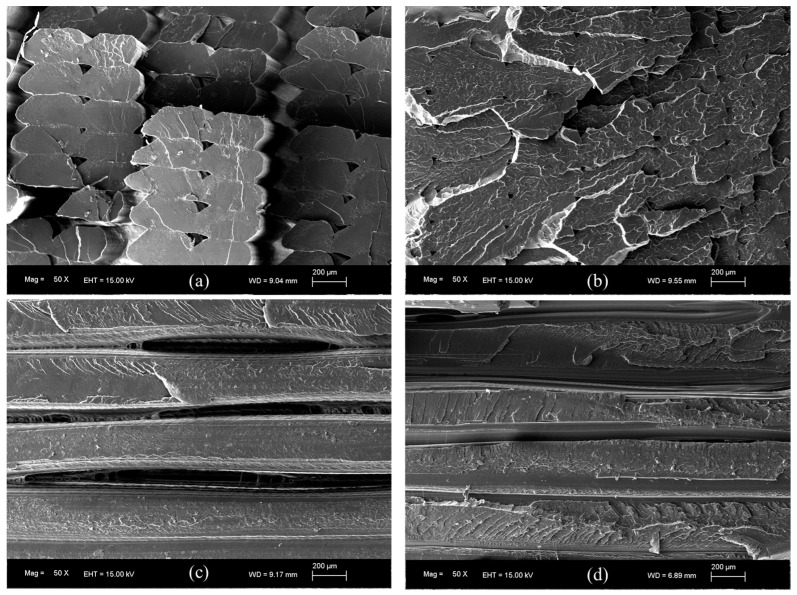
SEM images corresponding to the fracture surfaces of the (**a**,**b**) horizontal and the (**c**,**d**) transversal tensile specimens, printed at (**a**,**c**) 180 °C and (**b**,**d**) 240 °C.

The obtained values for the tensile strength and the tensile modulus are in good agreement with those presented in other works [40,41] and higher than the values reported in some other studies [42,43,44]. As can be seen in Table S6, the ANOVA demonstrates no significant variations in both the tensile strength and the tensile modulus when the nozzle temperature is increased from 200 to 240 °C. The maximum value for the tensile strength is in the 57.02–58.65 MPa range, obtained for a 200–240 °C nozzle temperature interval. For the same temperature interval, the tensile modulus reaches maximum values in the 3495.12–3714.28 MPa range. For all parameters, the lowest values correspond to the nozzle temperature of 180 °C. In order to explain this last phenomenon, the surface of the printed samples were examined. As can be seen in Figure 8a, the specimens printed at 180 °C are not completely solid and present some air gaps. It must be taken into account that the infill parameter of 100% was maintained constant for all temperatures during printing. The presence of these air gaps could be related to a high viscosity, restricting the flow of PLA during manufacturing. That hypothesis is supported by the weight measurements once pieces were printed, being the lowest values for these samples, and the SEM image shown in Figure 9a. As can be seen, for the samples printed at 180 °C, an absence of material is clearly, detected together with triangular voids between adjacent layers, which produces the lowest registered tensile strength and modulus values. This demonstrates that, if nozzle temperature is reduced too much, the infill density could decrease. It can be considered that at this temperature PLA is not heated enough to extrude correctly. Hsueh et al. [45] attributed the differences observed in the stress–strain curves for the 180 °C printed samples to an incomplete melting of the material. An increase in the nozzle temperature at 200 °C (see Figure 8b) gives rise to a good printing of the specimens, which corresponds to an increase in all mechanical properties, also observed by Hsueh et al. [45]. This increase in nozzle temperature is fundamental for good adherence of PLA layers. In fact, according to Figure 9b, the air gaps and the triangular voids were decreased drastically in the samples printed at higher temperatures, indicating a better infill with a good adhesion between layers, which is related to the increase in the mechanical parameters. However, if nozzle temperature is very high, an excess of material could be deposited, giving rise to a negative air gap, where two adjacent layers are overlapped, as can be seen in Figure 8c, decreasing the mechanical properties. This effect is supported by the results presented in Figure 7 and Table 2, where a decrease can be observed in the tensile strength and the tensile modulus values at 260 °C. In the case of the elongation at break, there is no evidence of changes from 200 to 260 °C extruder temperature, as can be seen in both Figure 7 and the ANOVA study (Table S6), where differences in the elongation at break cannot be assessed.

Comparing with other works, Tymrak et al. [40] measured, for 0.2 mm layer height specimens, maximum values of 60.4 MPa and 3480 MPa for tensile strength and tensile modulus, respectively. Similar values were obtained by Ferreira et al. [41], reporting 54.7 MPa for tensile strength and 3376 MPa for tensile modulus with a 190 °C extrusion temperature and layer height of 0.3 mm. Curiously, authors reported that printing at the recommended temperature by the manufacturer (220 °C) produced small bubble-like structures in the specimens. This problem was solved by printing at a lower temperature of 190 °C, i.e., in the middle of the 180–200 °C temperatures here studied, where material shortage is observed. Pastor et al. [43] obtained values of 29.82–31.55 MPa and 2063.6–2776.4 MPa for tensile strength and tensile modulus, respectively, with a nozzle temperature of 205 °C and a rectangular raster angle of 45°. Alafaghani et al. [42] studied different building configurations, infill, and a nozzle temperature interval of 175–205 °C. They reported 28.59–46.06 MPa and 37 1947.05–3177.53 MPa for tensile strength and tensile modulus, respectively. Webbe et al. [44] varied the infill density and the layer thickness, printing the specimens at 300 °C, obtaining 23.63–34.74 MPa and 526.38–1030.97 MPa for tensile strength and tensile modulus, respectively.

**Figure 10:**
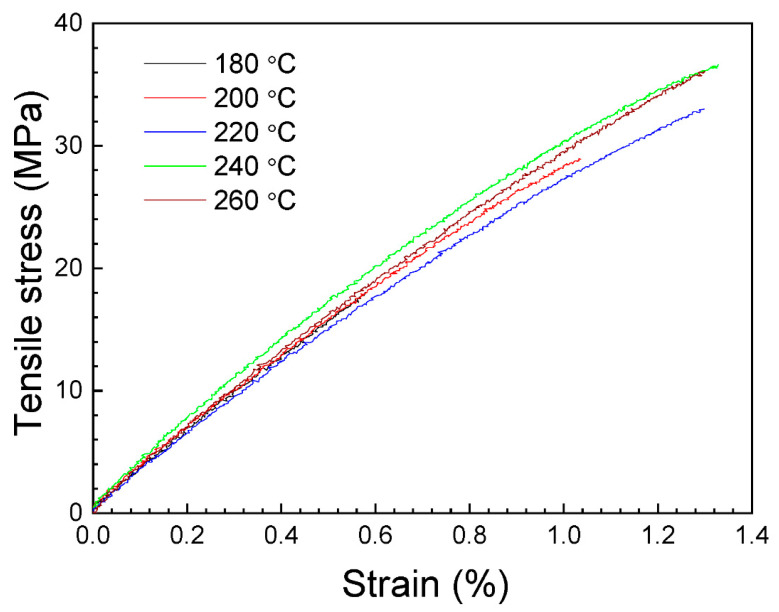
Representative stress–strain curves for the transversal manufactured samples.

**Figure 11:**
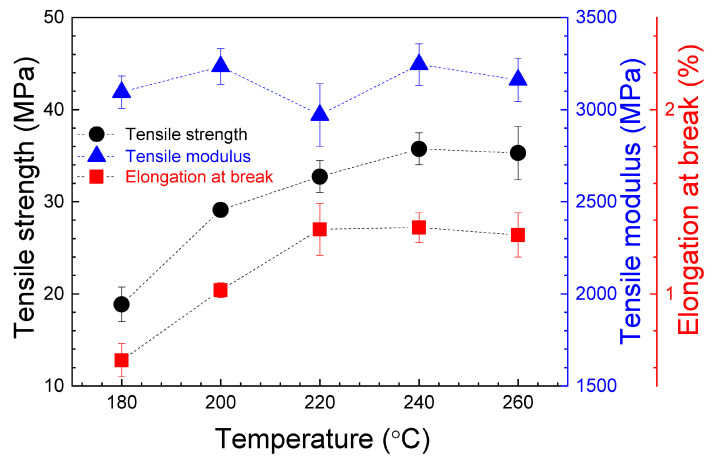
Average values of the tensile strength, the tensile modulus and the elongation at break, for the transversal manufactured tensile specimens, as a function of nozzle temperature. The error bars correspond to the standard deviations. The dashed lines are visual guides.

The representative stress–strain curves corresponding to the tensile tests of the transversal manufactured samples are presented in Figure 10. Figure 11 shows the relative dependence of the tensile strength, the tensile modulus and the elongation at break on nozzle temperature, and the average values, with their standard deviation, are presented in Table 3. As can be seen, the response is very similar to the first tested series (horizontally manufactured), but with three important differences: a less plastic zone before failure, lower values for tensile strength and lower percentage of strains at break. These results indicate that the transversal printed samples are more brittle with respect to the horizontal specimens. This behavior has also been reported in the literature by other authors, such as Corapi et al. [20]. The lowest values for tensile strength and tensile modulus are in agreement with the breaking form. In these transversal specimens, failure takes place along with the raster printing through raster-to-raster bonding, which is weaker than the individual raster (see Figures S4 and S5). In other words, the breaking in the horizontal configuration takes place along the deposited material whereas, for the transversal specimens, the breaking is due to the separation between layers, resulting in lower values for the studied mechanical properties.

In this configuration, when nozzle temperature increases, the tensile strength and the elongation at break increase from 180 °C up to maximum values in the 220–260 °C range. At this interval, there is no statistically significant difference, as demonstrated by the ANOVA presented in Table S7. This enhancement of tensile strength indicates that a higher temperature provides an increase in strength with respect to samples manufactured at a lower temperature, as successive layers will result in more cohesive properties. These results are in contrast with the behavior observed in horizontal printed specimens, where the maximum values for these parameters was reached in the 200–240 °C range. This increase reported in specimens with a transversal printing orientation could evidence that this printing orientation supports a higher nozzle temperature. According to SEM images (Figure 9c,d), for the samples printed at 180 °C, an absence of material, in the form of air gaps between layers, can be observed. When temperature increases, no air gaps are presented, which could be associated with an increase in mechanical properties. However, as can be seen in Figure 11, and supported by the ANOVA evaluation in Table S7, from 220 to 260 °C, stable maximum values in the ranges of 32.73–35.30 MPa and 1.32–1.35% are reported for tensile strength and elongation at break, respectively. With respect to the tensile modulus, according to Figure 11, a data oscillation was obtained, which could represent no changes. Nozzle temperature has no clear evident effect on this parameter when the pieces are printed along a transversal orientation.

### 3.3. Flexural Tests

**Figure 12:**
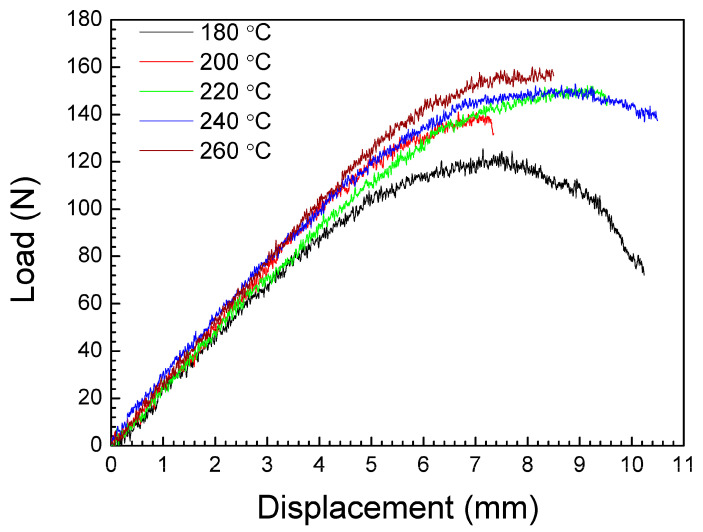
Representative load–displacement curves of the flexural specimens.

**Figure 13:**
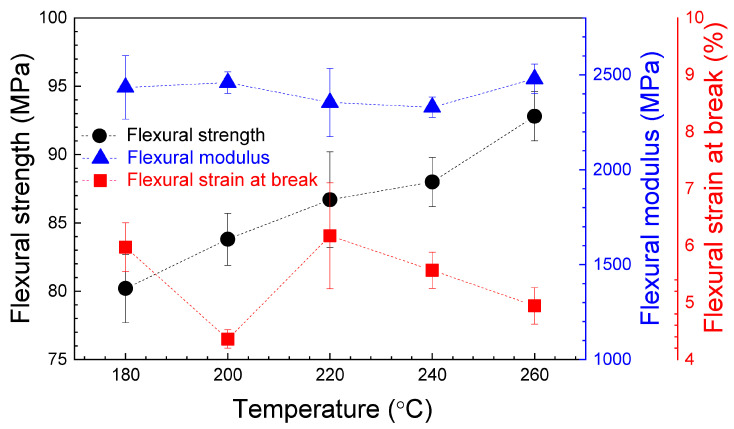
Average values of the flexural strength, the flexural modulus and the flexural strain at break, for the flexural specimens, as a function of nozzle temperature. The error bars correspond to the standard deviations. The dashed lines are visual guides.

Figure 12 shows representative load–displacement curves corresponding to the experimental flexural tests. As can be seen, all samples present an initial quasi-linear increase in load as a function of displacement, followed by a plastic region before breaking, with a maximum flexural load close to, or with the same value as, the flexural load at break. Some specimens, such as those printed at 180 and 220 °C, show a clear yield deflection curve, with both a yield and a break point. Figure 13 shows the relative dependence of the flexural strength, the flexural modulus and the elongation at break on nozzle temperature. The average values of these parameters, with their standard deviation, are presented in Table 4.

**Figure 14:**
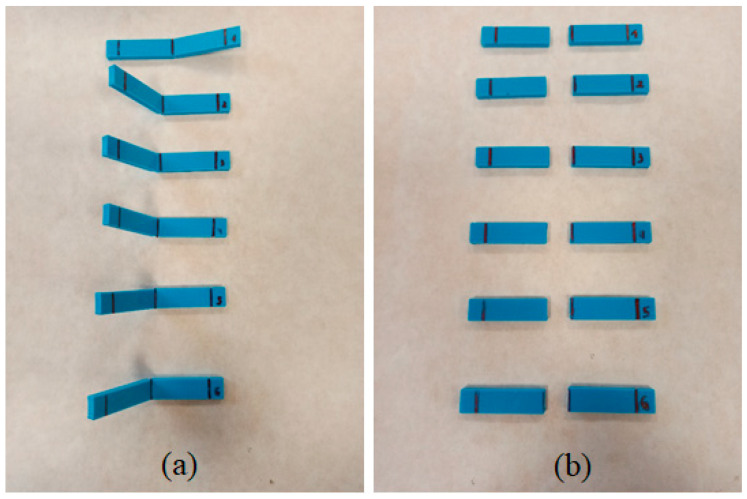
Images of the flexural tested specimens manufactured at nozzle temperatures of (**a**) 180 °C and (**b**) 260 °C.

**Figure 15:**
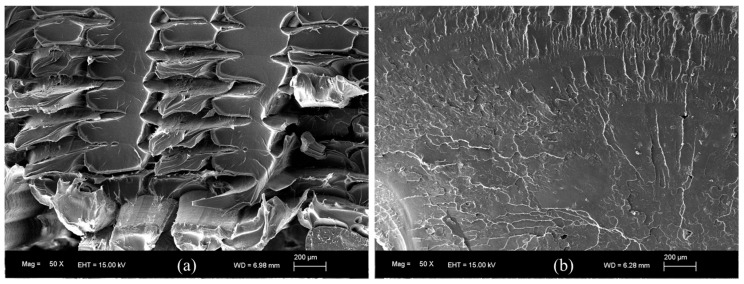
SEM images corresponding to the fracture surfaces of the flexural specimens printed at (**a**) 180 °C and (**b**) 240 °C.

It is to be noted that the flexural behavior of the printed sample at 180 °C (Figure 12) is quite different to the other samples. At the end of the tests, all these specimens (manufactured at 180 °C) slipped between the supports and did not break (see Figure 14a). The high value of the elongation at break observed for these samples (Figure 13 and Table 4) has no significant importance, and cannot be considered as an authentic material response under flexural loading. According to the SEM image in Figure 15a, the samples printed at 180 °C present air gaps between layers, which could explain the negative effects on mechanical properties.

As can be seen in Figure 14b, for the rest of the specimens, the breakage of the samples is produced along their longitudinal axis under the flexural stress load. The failure is observed across the layers, with no delamination on the interface between layers (see Figure S6). The initial crack started to appear at the bottom of the sample, located at the opposite side of the applied load, which it under tensile pressure. During the test, the samples are unbroken by the side of the rasters located on the compression side.

From Figure 13, Table 4 and the ANOVA in Table S8, the maximum value for the flexural strength is 92.8 MPa, corresponding to the samples printed at 260 °C. For the flexural modulus, the maximum value is in the 2329.86–2479.02 MPa interval, obtained for a 200–260 °C extruder temperature range. The flexural strain at break reaches the maximum values of 5.57–6.17% for the samples manufactured at 220–240 °C. The difference between the elastic modulus values obtained from tensile and flexural tests are a consequence of the difference in the mechanical response of the pieces under uniaxial force or blending at the middle point. In fact, in the blending configuration, the outer surface is under tensile pressure, whereas the surface where the force is applied is under compression stress. It is noted that a high nozzle temperature could have a great impact on flexural strength and flexural modulus. This could explain why the maximum values for these parameters include the highest nozzle temperature of 260 °C, in contrast with the tensile strength and the tensile modulus, where the maximum values are reached at lower nozzle temperatures. These results could be associated with a better adhesion of the deposited material during manufacturing. This conclusion is supported by the SEM image in Figure 15b where, at 240 °C of nozzle temperature, the air gaps observed at low printing temperature have disappeared. This corresponds to a higher infill, improving the cohesion between layers.

Compared with other works, similar behavior of flexural modulus is reported by Hsueh et al. [45] and with good agreement for flexural strength values (between 75–90 MPa). Pastor et al. [43] reported a similar 2.27–2.9 GPa for flexural modulus and lower values of 55.22–67.26 MPa for flexural strength. Maximum values of 79.02 MPa and 2.42 GPa were obtained for flexural strength and flexural modulus, respectively, for specimens printed at 200 °C, in a previous work [46]. A slightly lower flexural modulus of 2.13 GPa was registered by Nugroho et al. [47], with a flexural strength of 59.6 MPa.

### 3.4. Compression Tests

**Figure 16:**
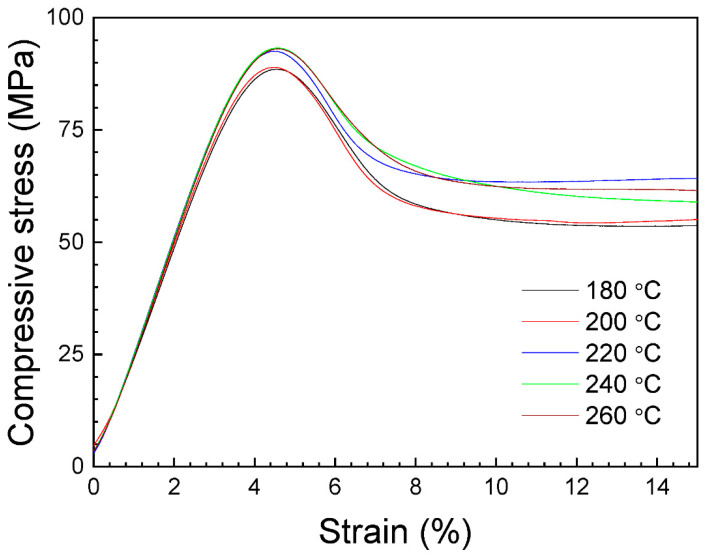
Representative stress–strain curves of the compression specimens.

Figure 16 shows representative stress–strain curves obtained from the experimental compression tests. The curves have a first linear region, corresponding to an elastic response to the compression load. This region is followed by an almost constant stress (plateau) when the strain increases. During this stage, the holes produced previously are being filled by material.

Under load, the tests can reach the maximum value of the machine applied force and, in our case, a limit of 45 kN was imposed. The complete stress–strain curves can be seen in Figure S7, where a third final region with an exponential rising in stress is observed. In fact, the samples continue to deform under load in compression until a flat disk is produced (see Figure S8). This rise can be attributed to a densification material process, in which the sample collapsed itself due to the applied force, reinforcing and increasing the stress. For this reason, the compressive yield strength will be considered as the maximum compressive stress in this work.

**Figure 17:**
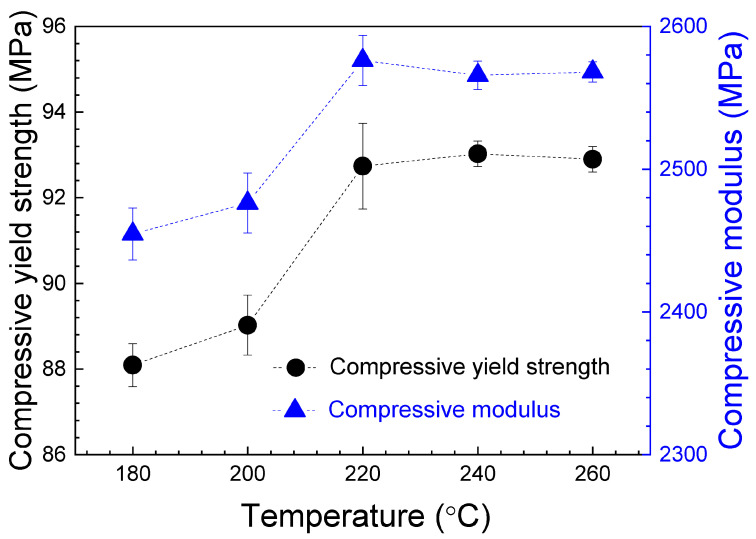
Average values of the compressive yield strength and the compressive modulus, for the compression specimens, as a function of nozzle temperature. The error bars correspond to the standard deviations. The dashed lines are visual guides.

Figure 17 shows the average values of the compressive yield strength and the compressive modulus on nozzle temperature. As can be seen, when the nozzle temperature increases, both the compressive yield strength and the compressive modulus slightly increase up to 220 °C. This increase in properties could be associated to a better adhesion between layers with printing temperature. However, from 220 to 260 °C, these values are approximately constant, and no increase is observed. Hsueh et al. [45] obtained, for PLA specimens under compression tests, the same behavior for both the compressive modulus and the compressive yield strength when increasing the nozzle temperature from 180 to 220 °C.

The average values of these parameters, with their corresponding standard deviation, are presented in Table 5. Taking into account the ANOVA study, presented in Table S9, the maximum value for the compressive yield strength is in the 92.7–93 MPa range, and for the compressive modulus in the 2565.78–2576.22 MPa range at a printing temperature interval of 220–260 °C. The difference between the elastic modulus values obtained from tensile and compression tests evidence the difference in mechanical behavior between these configurations. The compressive modulus values are lower than those obtained for tensile modulus. This behavior was also observed by Brischetto et al. [48], measuring, for samples printed at 215 °C, Young modulus of 2549.03 MPa and 2035.01 MPa from tensile and compression tests, respectively. The discrepancy of about 500 MPa between both tests is similar to that reported in this research with respect to the tensile transversal specimens. This fact is very important and must be considered in the fabrication of specimens, because PLA prototypes manufactured by FDM can present important mechanical differences depending on the tensile or compression configuration.

## 4. Conclusions

PLA specimens were fabricated by 3D printing FDM, varying the nozzle temperature from 180 to 260 °C. From XRD analysis, an amorphous structure is observed, and no crystallization is presented after printing, independently of the manufacturing temperature. Because no internal changes were observed, the differences in the mechanical properties can be attributed to the effect of the material flow and cohesion of rasters, as a consequence of the variation in the temperature.

If nozzle temperature is very low, PLA is not heated enough to fluid adequately. The presence of air gaps weakens the adhesion between layers, significantly affecting the mechanical properties of the sample. On the contrary, when the nozzle temperature increases too much, the extruded filament becomes more fluid, causing an overlapping of adjacent layers, but, in general, with no significant effect on mechanical properties. Only in some cases was a decrease in mechanical parameters observed.

From tensile tests, the highest values for tensile strength are in the 57.02–58.65 MPa range, obtained in the samples horizontally printed at the 200–240 °C nozzle temperature interval. For the same temperature range, the tensile modulus reaches the maximum value in the 3495.12–3714.28 MPa range. The elongation at break has no significant changes from 200 to 260 °C extruder temperature. Instead, for transversal orientation samples, the highest values for tensile strength and the elongation at break correspond to the samples printed from 220 to 260 °C, for which maximum values are in the 32.73–35.74 MPa and 1.32–1.35% ranges, respectively. The tensile modulus values show no evident differences in the studied temperature interval. For all parameters, the mean values are lower than that found for horizontal printed samples.

For bending tests, the maximum value for the flexural strength is 92.8 MPa, corresponding to the samples printed at 260 °C. For the flexural modulus, the maximum value is in the 2329.86–2479.02 MPa range, corresponding to the 200–260 °C nozzle temperature interval. The flexural strain at break reaches the highest values of 5.57–6.17% for the samples printed in the 220–240 °C temperature range. For the flexural test, the optimum printing temperature could be 220–260 °C.

With respect to the compression test, the maximum value for the compressive yield strength is in the 92.7–93 MPa range, and for the compressive modulus in the 2565.78–2576.22 MPa range, at a printing temperature interval of 220–260 °C.

In conclusion, taking into account all test information, an optimal nozzle interval could be 220–240 °C, which covers all parameters and avoids the highest temperature where an excess of material was detected. However, the selection of printing temperature will depend on the purpose of the required piece. For this reason, it is very important to consider if the specimen will be under tensile, flexural or compression effort. This could require a study of the demanded mechanical properties before selecting the manufacturing temperature. It must be noted that the obtained results are specific to the printer model employed in this work, the design of the specimens and the manufacturing parameters.

## Figures and Tables

**Table 1:** Fixed printing parameters.

Parameter	Value
Layer width	0.4 mm
Layer height	0.2 mm
Infill density	100%
Bed temperature	60 °C
Deposition speed: perimeters	40 mm/s
Deposition speed: infill	50 mm/s
Deposition speed: first layer	20 mm/s

**Table 2:** Average mechanical properties of the tensile tests for the horizontal manufactured specimens. The values in brackets correspond to the standard deviations.

Tensile Specimens—Horizontal Manufactured Orientation					
Nozzle Temperature (°C)	Tensile Strength (MPa)	Tensile Modulus (MPa)	Elongation at Break (%)	Ambient Conditions	
				T (°C)	HR (%)
180	45.50 (2.21)	3220.46 (54.65)	2.04 (0.22)	18.1	66.3
200	58.65 (1.84)	3495.12 (86.74)	2.49 (0.33)	17.9	61.7
220	57.64 (2.17)	3660.66 (279.78)	2.31 (0.22)	17.6	61.0
240	57.02 (1.88)	3714.28 (159.88)	2.50 (0.35)	17.7	66.0
260	51.71 (1.74)	3332.62 (122.92)	2.19 (0.23)	17.1	72.1

**Table 3:** Average mechanical properties of the tensile tests for transversal manufactured specimens. The values in brackets correspond to the standard deviations.

Tensile Specimens—Transversal Manufactured Orientation					
Nozzle Temperature (°C)	Tensile Strength (MPa)	Tensile Modulus (MPa)	Elongation at Break (%)	Ambient Conditions	
				T (°C)	HR (%)
180	18.86 (1.88)	3095.08 (87.49)	0.64 (0.09)	20.3	66.0
200	29.11 (0.79)	3233.96 (97.17)	1.02 (0.04)	19.2	65.5
220	32.73 (1.74)	2970.50 (170.15)	1.35 (0.14)	20.2	68.4
240	35.74 (1.74)	3245.00 (112.52)	1.36 (0.08)	20.3	67.8
260	35.30 (2.90)	3161.10 (117.89)	1.32 (0.12)	19.1	64.8

**Table 4:** Average mechanical properties of flexural tests. The values in brackets correspond to the standard deviations.

Flexural Specimens					
Nozzle Temperature (°C)	Flexural Strength (MPa)	Flexural Modulus (MPa)	Flexural Strain at Break (%)	Ambient Conditions	
				T (°C)	HR (%)
180	80.2 (2.5)	2434.41 (167.22)	5.97 (0.43)	16.9	61.0
200	83.8 (1.9)	2459.10 (57.15)	4.36 (0.16)	17.2	63.9
220	86.7 (3.5)	2354.80 (179.49)	6.17 (0.93)	17.6	62.4
240	88.0 (1.8)	2329.86 (53.88)	5.57 (0.32)	17.7	66.4
260	92.8 (1.8)	2479.02 (77.33)	4.97 (0.32)	17.7	65.0

**Table 5:** Average mechanical properties of compression tests. The values in brackets correspond to the standard deviations.

Compressive Specimens				
Nozzle Temperature (°C)	Compressive Yield Strength (MPa)	Compressive Modulus (MPa)	Ambient Conditions	
			T (°C)	HR (%)
180	88.1 (0.5)	2454.58 (18.20)	17.9	63.0
200	89.0 (0.7)	2476.28 (21.01)	18.4	62.0
220	92.7 (1.0)	2576.22 (17.57)	17.7	55.0
240	93.0 (0.3)	2565.78 (9.96)	17.7	62.3
260	92.9 (0.3)	2568.16 (7.08)	18.0	67.7

## Data Availability

The original contributions presented in the study are included in the article/Supplementary Material, further inquiries can be directed to the corresponding author.

## Supplementary Materials

The following supporting information can be downloaded at: https://www.mdpi.com/article/10.3390/polym16131867/s1, Table S1: Basic information for the weights and dimensions of each horizontal manufactured specimen for tensile tests. The labels of the measured values correspond to the points indicated in Figure S1; Table S2: Basic information for the weights and dimensions of each transversal manufactured specimen for tensile tests. The labels of the measured values correspond to the points indicated in Figure S1; Table S3: Basic information for the weights and dimensions of each printed specimen for flexural tests. The labels of the measured values correspond to the points indicated in Figure S2; Table S4: Basic information for the weights and dimensions the printed specimen for compression tests. The labels of the measured values correspond to the points indicated in Figure S3; Table S5: Temperature and humidity mean values measured during manufacturing process; Table S6: ANOVA for tensile specimens—horizontal manufactured orientation; Table S7: ANOVA for tensile specimens—transversal manufactured orientation; Table S8: ANOVA for flexural specimens; Table S9: ANOVA for compressive specimens; Figure S1: Specimen for tensile test with the marks of the dimensional measurements; Figure S2: Specimen for flexural test with the marks of the dimensional measurements; Figure S3: Specimen for compression test with the marks of the dimensional measurements; Figure S4: Optical images of the fracture surface corresponding to the tensile tested horizontal printed specimens; Figure S5: Optical images of the fracture surface corresponding to the tensile tested transversal printed specimens; Figure S6: Optical images of the fracture surface corresponding to the flexural tested specimens; Figure S7: Representative of the complete stress-strain curves, until 45 kN of load, for the compression specimens; Figure S8: Optical images of the tested compression specimens.
