# Supplementary material for: Effects of Nozzle Temperature on Mechanical Properties of Polylactic Acid Specimens Fabricated by Fused Deposition Modeling
Source: Polymers (Basel). 2024 Jun 29;16(13):1867. doi: 10.3390/polym16131867 (PMC11243908; doi:10.3390/polym16131867)

Supplementary Information

Effects of Nozzle Temperature on Mechanical Properties of Polylactic Acid Specimens Fabricated by Fused Deposition Modeling

Fernando Rivera-López*, María M. Laz Pavón, Eduardo Cabello Correa, María Hernández Molina

Departamento de Ingeniería Industrial. Escuela Superior de Ingeniería y Tecnología. Universidad de La Laguna, Apdo. 456, E-38200 San Cristóbal de La Laguna, Santa Cruz de Tenerife, Spain.

* Correspondence: frivera@ull.es

Table S1. Basic information of the weights and dimensions of each horizontal manufactured specimen for tensile tests. The labels of the measured values correspond to the points indicated in Figure S1.

Tensile specimens horizontal manufactured			Dimensions (mm)															
Printing temperature (°C)	Specimen	Weight (g)	t1	t2	t3	t4	t5	t	W_{01}	W_{02}	W_0	W_1	W_2	W_3	W	l_3	L_{ext}
180	Specimen 1	10.32	3.98	3.96	3.96	3.94	3.94	3.96	20.06	20.11	20.08	10.07	10.08	10.10	10.08	170.00	50.00
	Specimen 2	10.29	3.96	3.95	3.94	3.93	3.94	3.94	20.11	20.09	20.10	10.07	10.09	10.10	10.09	170.00	50.00
	Specimen 3	10.26	3.96	3.98	3.97	3.96	3.93	3.96	20.13	20.10	20.11	10.09	10.10	10.11	10.10	170.00	50.00
	Specimen 4	10.28	3.95	3.93	3.94	3.96	3.97	3.95	20.10	20.10	20.10	10.08	10’1	10.13	10.10	170.00	50.00
	Specimen 5	10.31	3.98	3.98	4.00	3.98	3.96	3.98	20.13	20.14	20.13	10.13	10.12	10.17	10.14	170.00	50.00
	Specimen 6	10.35	3.98	3.98	3.99	3.97	4.02	3.99	20.07	20.08	20.07	10.14	10.15	10.14	10.14	170.00	50.00
200	Specimen 1	11.67	3.95	3.95	3.95	3.94	3.94	3.95	20.35	20.36	20.35	10.22	10.24	10.19	10.22	170.00	50.00
	Specimen 2	11.73	4.03	3.98	3.98	3.98	3.97	3.99	20.20	20.22	20.21	10.30	10.35	10.30	10.32	170.00	50.00
	Specimen 3	11.70	3.96	3.93	3.97	3.99	2.96	3.76	20.23	20.20	20.21	10.29	10.30	10.29	10.29	170.00	50.00
	Specimen 4	11.70	3.99	3.99	3.94	3.94	3.95	3.96	20.21	20.10	20.15	10.23	10.30	10.33	10.29	170.00	50.00
	Specimen 5	11.69	3.95	3.96	3.95	3.92	3.97	3.95	20.20	20.19	20.19	10.20	10.30	10.33	10.28	170.00	50.00
	Specimen 6	11.72	3.95	4.00	3.98	3.96	3.99	3.98	20.27	20.24	20.25	10.34	10.33	10.30	10.32	170.00	50.00
220	Specimen 1	11.86	3.97	3.93	3.95	3.94	3.96	3.95	20.15	20.16	20.15	10.19	10.20	10.22	10.20	170.00	50.00
	Specimen 2	11.85	4.01	3.95	3.97	3.97	4.01	3.98	20.20	20.30	20.25	10.40	10.40	10.28	10.36	170.00	50.00
	Specimen 3	11.89	3.98	4.00	3.97	3.98	3.98	3.98	20.24	20.30	20.27	10.32	10.27	10.31	10.30	170.00	50.00
	Specimen 4	11.90	4.04	3.95	3.98	4.05	3.98	4.00	20.30	20.21	20.25	10.30	10.40	10.45	10.38	170.00	50.00
	Specimen 5	11.91	3.98	4.04	4.01	4.05	4.04	4.02	20.40	20.35	20.37	10.48	10.42	10.39	10.43	170.00	50.00
	Specimen 6	11.94	4.07	4.06	4.03	3.97	4.00	4.03	20.35	20.25	20.30	10.30	10.31	10.35	10.32	170.00	50.00
240	Specimen 1	11.80	3.96	4.01	3.98	3.98	4.02	3.99	20.32	20.36	20.34	10.40	10.38	10.42	10.40	170.00	50.00
	Specimen 2	11.84	4.03	3.96	4.01	4.07	4.01	4.02	20.39	20.25	20.32	10.35	10.40	10.40	10.38	170.00	50.00
	Specimen 3	11.85	3.98	3.97	3.97	3.98	3.98	3.98	20.38	20.32	20.35	10.35	10.32	10.38	10.35	170.00	50.00
	Specimen 4	11.83	4.02	3.99	4.00	4.02	4.06	4.02	20.32	20.26	20.29	10.32	10.38	10.44	10.38	170.00	50.00
	Specimen 5	11.85	4.01	4.00	4.01	3.99	3.99	4.00	20.33	20.34	20.33	10.45	10.40	10.40	10.42	170.00	50.00
	Specimen 6	11.85	3.97	3.95	3.95	3.96	3.96	3.96	20.26	20.35	20.30	10.42	10.39	10.37	10.39	170.00	50.00
260	Specimen 1	11.96	3.99	4.07	3.96	4.09	4.02	4.03	20.82	20.86	20.84	10.88	10.91	10.93	10.91	170.00	50.00
	Specimen 2	11.94	4.02	4.09	4.04	4.09	4.16	4.08	20.76	20.78	20.77	10.89	10.80	10.83	10.84	170.00	50.00
	Specimen 3	11.91	4.00	4.01	3.95	3.94	4.05	3.99	20.75	20.76	20.75	10.95	10.95	10.98	10.96	170.00	50.00
	Specimen 4	11.93	4.12	4.05	3.97	4.01	4.11	4.05	20.78	20.81	20.79	10.92	10.88	10.90	10.90	170.00	50.00
	Specimen 5	11.94	3.99	3.96	4.02	4.10	3.99	4.01	20.78	20.86	20.82	10.94	10.89	10.98	10.94	170.00	50.00
	Specimen 6	11.98	4.19	3.97	4.10	4.06	4.14	4.09	20.68	20.76	20.72	10.93	10.88	10.87	10.89	170.00	50.00

Table S2. Basic information of the weights and dimensions of each transversal manufactured specimen for tensile tests. The labels of the measured values correspond to the points indicated in Figure S1.

Tensile speciemns tansversal manufactured			Dimensions (mm)														
**Printing temperature (°C)**	**Specimen**	**Weight (g)**	**t1**	**t2**	**t3**	**t4**	**t5**	**t**	**W_{01}**	**W_{02}**	**W_0**	**W_1**	**W_2**	**W_3**	**W**	**l_3**	**L_{ext}**
**180**	Specimen 1	11.79	4.40	4.36	4.15	4.17	4.22	4.26	20.41	20.35	20.38	10.34	10.31	10.33	10.33	170.00	50.00
	Specimen 2	11.71	4.38	4.12	4.11	4.10	4.16	4.17	20.42	20.33	20.37	10.30	10.28	10.30	10.29	170.00	50.00
	Specimen 3	11.72	4.27	4.15	4.12	4.11	4.17	4.16	20.32	20.39	20.35	10.24	10.30	10.30	10.28	170.00	50.00
	Specimen 4	11.74	4.28	4.12	4.14	4.14	4.14	4.16	20.33	20.36	20.34	10.28	10.29	10.28	10.28	170.00	50.00
	Specimen 5	11.74	4.31	4.13	4.15	4.13	4.16	4.18	20.34	20.37	20.35	10.24	10.29	10.21	10.25	170.00	50.00
	Specimen 6	11.75	4.35	4.13	4.15	4.11	4.18	4.18	20.36	20.34	20.35	10.24	10.27	10.27	10.26	170.00	50.00
**200**	Specimen 1	12.05	4.43	4.26	4.30	4.32	4.31	4.32	20.25	20.26	20.25	10.26	10.31	10.31	10.29	170.00	50.00
	Specimen 2	11.97	4.44	4.30	4.29	4.33	4.30	4.33	20.20	20.30	20.25	10.22	10.28	10.25	10.25	170.00	50.00
	Specimen 3	12.01	4.41	4.31	4.33	4.28	4.28	4.32	20.25	20.34	20.29	10.21	10.28	10.31	10.27	170.00	50.00
	Specimen 4	12.01	4.45	4.29	4.33	4.28	4.29	4.33	20.23	20.31	20.27	10.24	10.29	10.27	10.27	170.00	50.00
	Specimen 5	12.00	4.39	4.26	4.28	4.30	4.29	4.30	20.27	20.31	20.29	10.23	10.28	10.28	10.26	170.00	50.00
	Specimen 6	12.03	4.37	4.33	4.32	4.27	4.25	4.31	20.26	20.30	20.28	10.23	10.31	10.30	10.28	170.00	50.00
**220**	Specimen 1	12.12	4.45	4.30	4.27	4.28	4.38	4.34	20.46	20.41	20.43	10.39	10.39	10.41	10.40	170.00	50.00
	Specimen 2	11.98	4.39	4.28	4.25	4.25	4.29	4.29	20.42	20.41	20.41	10.38	10.34	10.40	10.37	170.00	50.00
	Specimen 3	12.06	4.34	4.33	4.35	4.26	4.29	4.31	20.44	20.45	20.44	10.40	10.37	10.38	10.38	170.00	50.00
	Specimen 4	12.06	4.37	4.31	4.27	4.27	4.26	4.30	20.49	20.46	20.47	10.39	10.39	10.41	10.40	170.00	50.00
	Specimen 5	12.06	4.44	4.29	4.27	4.30	4.30	4.32	20.45	20.44	20.44	10.38	10.37	10.42	10.39	170.00	50.00
	Specimen 6	12.07	4.39	4.27	4.25	4.25	4.28	4.29	20.45	20.43	20.44	10.40	10.35	10.39	10.38	170.00	50.00
**240**	Specimen 1	12.19	4.41	4.32	4.26	4.28	4.32	4.32	20.42	20.41	20.41	10.44	10.40	10.39	10.41	170.00	50.00
	Specimen 2	12.09	4.40	4.25	4.24	4.30	4.35	4.31	20.45	20.47	20.46	10.39	10.38	10.40	10.39	170.00	50.00
	Specimen 3	12.14	4.36	4.33	4.25	4.25	4.34	4.31	20.48	20.45	20.46	10.40	10.39	10.40	10.40	170.00	50.00
	Specimen 4	12.13	4.40	4.35	4.25	4.27	4.30	4.31	20.45	20.44	20.44	10.37	10.32	10.36	10.35	170.00	50.00
	Specimen 5	12.15	4.38	4.33	4.28	4.27	4.31	4.31	20.44	20.42	20.43	10.39	10.38	10.39	10.39	170.00	50.00
	Specimen 6	12.17	4.31	4.30	4.25	4.24	4.35	4.29	20.44	20.48	20.46	10.36	10.39	10.38	10.38	170.00	50.00
**260**	Specimen 1	12.26	4.39	4.35	4.29	4.27	4.30	4.32	20.44	20.46	20.45	10.40	10.40	10.41	10.40	170.00	50.00
	Specimen 2	12.25	4.32	4.29	4.30	4.22	4.35	4.30	20.34	20.42	20.38	10.36	10.38	10.40	10.38	170.00	50.00
	Specimen 3	12.26	4.36	4.29	4.25	4.28	4.35	4.31	20.38	20.47	20.42	10.40	10.39	10.38	10.39	170.00	50.00
	Specimen 4	12.28	4.39	4.28	4.25	4.29	4.39	4.32	20.45	20.43	20.44	10.39	10.37	10.36	10.37	170.00	50.00
	Specimen 5	12.28	4.40	4.31	4.26	4.25	4.38	4.32	20.41	20.49	20.45	10.38	10.39	10.40	10.39	170.00	50.00
	Specimen 6	12.31	4.35	4.22	4.25	4.29	4.38	4.30	20.40	20.48	20.44	10.38	10.36	10.39	10.38	170.00	50.00

Table S3. Basic information of the weights and dimensions of each printed specimen for flexural tests. The labels of the measured values correspond to the points indicated in Figure S2.

Flexural			Dimensions (mm)								
**Printing temperatura**	**Specimen**	**Weight (g)**	**b_1**	**b_2**	**b_3**	**b**	**h_1**	**h_2**	**h_3**	**h**	**l**
**180**	Specimen 1	3.62	10.56	10.66	10.65	10.62	3.83	3.85	3.87	3.85	80.00
	Specimen 2	3.61	10.61	10.69	10.61	10.64	3.92	3.85	3.91	3.89	80.00
	Specimen 3	3.62	10.55	10.61	10.56	10.57	3.95	3.89	3.90	3.91	80.00
	Specimen 4	3.62	10.59	10.74	10.60	10.64	3.88	3.90	3.93	3.90	80.00
	Specimen 5	3.63	10.60	10.74	10.64	10.66	3.92	3.91	3.94	3.92	80.00
	Specimen 6	3.62	10.60	10.75	10.62	10.66	3.93	3.92	3.96	3.94	80.00
**200**	Specimen 1	3.87	10.71	10.67	10.69	10.69	3.94	3.94	4.03	3.97	80.00
	Specimen 2	3.89	10.73	10.81	10.72	10.75	4.09	4.00	3.96	4.02	80.00
	Specimen 3	3.88	10.66	10.75	10.67	10.69	4.10	3.98	4.00	4.03	80.00
	Specimen 4	3.88	10.67	10.78	10.65	10.70	4.06	4.01	4.03	4.03	80.00
	Specimen 5	3.87	10.65	10.79	10.69	10.71	4.06	3.97	4.03	4.02	80.00
	Specimen 6	3.87	10.72	10.80	10.72	10.75	4.05	4.07	4.06	4.06	80.00
**220**	Specimen 1	3.89	10.75	10.85	10.86	10.82	4.00	4.02	4.05	4.02	80.00
	Specimen 2	3.88	10.75	10.78	10.73	10.75	4.16	4.07	4.02	4.08	80.00
	Specimen 3	3.89	10.74	10.77	10.69	10.73	4.16	4.08	4.05	4.10	80.00
	Specimen 4	3.89	10.79	10.81	10.77	10.79	4.14	4.02	4.04	4.07	80.00
	Specimen 5	3.89	10.80	10.73	10.66	10.73	3.97	3.92	4.04	3.98	80.00
	Specimen 6	3.89	10.83	10.76	10.66	10.75	3.96	4.03	4.08	4.02	80.00
**240**	Specimen 1	3.96	10.78	10.92	10.79	10.83	4.04	3.99	4.04	4.02	80.00
	Specimen 2	3.97	10.76	10.84	10.79	10.80	4.09	4.00	4.01	4.03	80.00
	Specimen 3	3.96	10.68	10.84	10.68	10.73	4.08	3.96	4.01	4.02	80.00
	Specimen 4	3.96	10.75	10.86	10.76	10.79	4.08	4.02	4.06	4.05	80.00
	Specimen 5	3.95	10.81	10.88	10.82	10.84	4.05	4.00	4.08	4.04	80.00
	Specimen 6	3.95	10.70	10.82	10.73	10.75	4.02	3.99	4.08	4.03	80.00
**260**	Specimen 1	3.96	10.84	10.91	10.86	10.87	4.01	4.00	4.07	4.03	80.00
	Specimen 2	3.96	10.74	10.83	10.70	10.76	4.01	4.02	4.03	4.02	80.00
	Specimen 3	3.95	10.73	10.85	10.72	10.77	4.04	4.03	4.04	4.04	80.00
	Specimen 4	3.95	10.76	10.82	10.75	10.78	4.11	4.00	4.08	4.06	80.00
	Specimen 5	3.95	10.79	10.87	10.78	10.81	4.09	4.04	4.02	4.05	80.00
	Specimen 6	3.96	10.77	10.88	10.74	10.80	4.07	4.05	4.02	4.05	80.00

Table S4. Basic information of the weights and dimensions the printed specimen for compression tests. The labels of the measured values correspond to the points indicated in Figure S3.

Compression			Dimensions (mm)												
**Printing temperature (°C)**	**Specimen**	**Weight (g)**	**e_1**	**e_2**	**e_3**	**e**	**d_1**	**d_2**	**d_3**	**d**	**L_1**	**L_2**	**L_3**	**L_4**	**L**
**180**	Specimen 1	12.14	15.77	15.80	15.82	15.80	16.04	16.06	16.04	16.05	39.86	39.94	40.16	39.98	39.99
	Specimen 2	12.10	15.76	15.76	15.75	15.76	16.08	16.16	16.12	16.12	39.84	39.99	39.91	39.88	39.91
	Specimen 3	12.10	15.77	15.76	15.77	15.77	16.16	16.11	16.22	16.16	39.84	39.92	39.83	39.92	39.88
	Specimen 4	12.13	15.76	15.76	15.80	15.77	16.14	16.16	16.14	16.15	39.86	39.94	40.16	39.91	39.97
	Specimen 5	12.15	15.78	15.78	15.80	15.79	16.11	16.12	16.16	16.13	39.90	39.92	40.11	39.92	39.96
	Specimen 6	12.14	15.74	15.74	15.78	15.75	16.08	16.11	16.15	16.11	39.91	39.98	39.85	39.88	39.91
**200**	Specimen 1	12.29	15.77	15.75	15.78	15.77	16.40	16.60	16.60	16.53	40.10	40.00	40.58	40.03	40.18
	Specimen 2	12.30	15.80	15.82	15.84	15.82	16.56	16.62	16.61	16.60	39.98	39.90	40.29	40.30	40.12
	Specimen 3	12.30	15.81	15.80	15.84	15.82	16.48	16.54	16.40	16.47	40.11	40.00	40.31	40.02	40.11
	Specimen 4	12.32	15.83	15.86	15.87	15.85	16.54	16.51	16.64	16.56	40.15	40.00	40.30	40.03	40.12
	Specimen 5	12.32	15.84	15.86	15.87	15.86	16.52	16.53	16.52	16.52	39.90	40.03	40.30	40.01	40.06
	Specimen 6	12.34	15.88	15.85	15.91	15.88	16.55	16.50	16.53	16.53	40.09	40.02	40.24	40.03	40.10
**220**	Specimen 1	12.64	15.80	15.88	15.96	15.88	16.16	16.32	16.44	16.31	39.98	40.05	40.21	40.01	40.06
	Specimen 2	12.65	15.64	15.71	15.80	15.72	16.36	16.32	16.34	16.34	40.06	40.09	40.36	40.11	40.16
	Specimen 3	12.66	15.83	15.89	15.93	15.88	16.41	16.40	16.48	16.43	40.06	40.05	40.26	40.02	40.10
	Specimen 4	12.65	15.76	15.88	15.91	15.85	16.32	16.38	16.38	16.36	40.10	40.11	40.33	40.09	40.16
	Specimen 5	12.64	15.78	15.82	15.92	15.84	16.23	16.35	16.36	16.31	40.10	40.23	40.36	40.08	40.19
	Specimen 6	12.62	15.79	15.86	15.93	15.86	16.17	16.24	16.35	16.25	40.13	40.10	40.36	40.12	40.18
**240**	Specimen 1	12.56	15.86	15.84	15.85	15.85	16.14	16.20	16.31	16.22	40.03	40.01	40.43	40.08	40.14
	Specimen 2	12.57	15.83	15.89	15.87	15.86	16.23	16.28	16.30	16.27	40.01	40.05	40.44	40.09	40.15
	Specimen 3	12.58	15.77	15.88	15.80	15.82	16.37	16.34	16.33	16.35	40.05	40.15	40.45	40.04	40.17
	Specimen 4	12.57	15.77	15.79	15.79	15.78	16.24	16.27	16.27	16.26	40.05	40.17	40.42	40.14	40.20
	Specimen 5	12.58	15.79	15.83	15.84	15.82	16.18	16.23	16.32	16.24	40.08	40.14	40.41	40.13	40.19
	Specimen 6	12.56	15.81	15.89	15.88	15.86	16.21	16.25	16.30	16.25	40.09	40.14	40.54	40.07	40.21
**260**	Specimen 1	12.74	15.86	15.86	15.92	15.88	16.27	16.31	16.32	16.30	40.04	40.11	40.45	40.15	40.19
	Specimen 2	12.74	15.90	15.84	15.89	15.88	16.27	16.30	16.36	16.31	40.20	40.21	40.50	40.15	40.27
	Specimen 3	12.75	15.81	15.79	15.83	15.81	16.29	16.34	16.37	16.33	40.15	40.13	40.58	40.14	40.25
	Specimen 4	12.75	15.82	15.93	15.91	15.89	16.25	16.28	16.33	16.29	40.13	40.10	40.56	40.07	40.22
	Specimen 5	12.75	15.87	15.84	15.89	15.87	16.28	16.30	16.33	16.30	40.18	40.09	40.51	40.07	40.21
	Specimen 6	12.74	15.79	15.80	15.89	15.83	16.28	16.28	16.29	16.28	40.24	40.13	40.35	40.10	40.21

Table S5. Temperature and humidity mean values measured during manufacturing process.

			180 °C		200 °C		220 °C		240 °C		260 °C	
			T (°C)	HR (%)	T (°C)	HR (%)	T (°C)	HR (%)	T (°C)	HR (%)	T (°C)	HR (%)
3D Samples	XRD		16.8	50.0	-	-	-	-	-	-	17.5	46.0
	Tensile	Horizontal manufactured	17.8	64.2	17.4	63.7	17.6	59.1	17.1	59.8	16.9	74.0
		Tansversal manufactured	18.6	58.6	19.1	61.7	18.9	62.2	19.1	61.9	19.1	58.9
	Compression		18.1	61.7	17.2	65.3	17.8	62.9	17.6	62.4	17.6	55.0
	Flexural		17.7	59.2	17.1	59.3	17.0	60.3	17.3	64.0	17.6	62.1

Table S6. ANOVA for tensile specimens - horizontal manufactured orientation.

Tensile strength

Temperatures	Sum of squares	Mean square	p-value	F-value	F-critical
180 - 200	432.3589	432.3589	$7.16x10^{-6}$	104.6494	5.3177
200 - 220	2.5523	2.5523	0.4505	0.6292	5.3177
220 - 240	0.9560	0.9560	0.6429	0.2321	5.3177
240 - 260	70.5912	70.5912	0.0017	21.5565	5.3177
200-220-240	6.7605	3.3803	0.4432	0.8715	3.8853

Tensile modulus

Temperatures	Sum of squares	Mean square	p-value	F-value	F-critical
180 - 200	188595.289	188595.289	0.0003	35.8880	5.3177
200 - 220	68508.729	68508.729	0.2419	1.5969	5.3177
220 - 240	7187.761	7187.761	0.7195	0.1384	5.3177
240 - 260	364160.889	364160.889	0.0029	17.9076	5.3177
200-220-240	130516.169	65258.0847	0.2140	1.7580	3.8853

Elongation at break

Temperatures	Sum of squares	Mean square	p-value	F-value	F-critical
180 - 200	0.5018	0.5018	0.0344	6.4777	5.3177
200 - 220	0.0774	0.0774	0.3503	0.9837	5.3177
220 - 240	0.0884	0.0884	0.3352	1.0512	5.3177
240 - 260	0.2372	0.2372	0.1336	2.7865	5.3177
200-220-240-260	0.3292	0.1097	0.2967	1.3395	3.2389

Table S7. ANOVA for tensile specimens - transversal manufactured orientation.

Tensile strength

Temperatures	Sum of Squares	Mean Square	p-value	F-value	F-critical
180 - 200	261.121	261.121	6.1756×10^{-6}	108.8685	5.3177
200 - 220	33.124	33.124	0.0060	13.6933	5.3177
220 - 240	22.5	22.5	0.0552	5.0302	5.3177
240 - 260	0.529	0.529	0.7896	0.0762	5.3177
220-240-260	26.1053	13.0527	0.1488	2.2423	3.8853

Tensile modulus

Temperatures	Sum of Squares	Mean Square	p-value	F-value	F-critical
180 - 200	48219.136	48219.136	0.0338	6.5392	5.3177
200 - 220	173527.929	173527.929	0.0085	12.0259	5.3177
220 - 240	188375.625	188375.625	0.0149	9.5346	5.3177
240 - 260	17598.025	17598.025	0.3224	1.1122	5.3177

Elongation at break

Temperatures	Sum of Squares	Mean Square	p-value	F-value	F-critical
180 - 200	0.361	0.361	0.0001	48.1333	5.3177
200 - 220	0.256	0.256	0.0019	20.48	5.3177
220 - 240	0	0	1	0	5.3177
240 - 260	0.001	0.001	0.8028	0.0667	5.3177
220-240-260	0.0013	0.0007	0.9631	0.0377	3.8853

Table S8. ANOVA for flexural specimens.

Flexural strength

Temperatures	Sum of Squares	Mean Square	p-value	F-value	F-critical
180 - 200	33.124	33.124	0.0304	6.8915	5.3177
200 - 220	20.164	20.164	0.1540	2.4794	5.3177
220 - 240	4.356	4.356	0.4804	0.5479	5.3177
240 - 260	57.6	57.6	0.0032	17.1556	5.3177
200-220-240	45.1893	22.5947	0.0648	3.4672	3.8853

Flexural modulus

Temperatures	Sum of Squares	Mean Square	p-value	F-value	F-critical
180 - 200	1523.9903	1523.9903	0.7627	0.0976	5.3177
200 - 220	27196.225	27196.225	0.2508	1.5330	5.3177
220 - 240	1580.3004	1580.3004	0.7719	0.0899	5.3177
240 - 260	55768.0368	55768.0368	0.0076	12.5290	5.3177
180-200-220-240	57732.714	19244.238	0.3557	1.1599	3.2389
200-220-240-260	82998.4268	27666.1423	0.0971	2.4933	3.2389

Flexural strain at break

Temperatures	Sum of Squares	Mean Square	p-value	F-value	F-critical
180 - 200	6.561	6.561	3.0107×10^{-6}	70.9297	5.3177
200 - 220	8.1	8.1	0.0027	18.1818	5.3177
220 - 240	0.841	0.841	0.2244	1.7340	5.3177
240 - 260	0.9	0.9	0.0179	8.8235	5.3177

Table S9. ANOVA for compressive specimens.

Compressive yield strength

Temperatures	Sum of Squares	Mean Square	p-value	F-value	F-critical
180 - 200	2.209	2.209	0.0264	7.3756	5.3177
200 - 220	34.596	34.596	9.6224×10^{-6}	51.2533	
220 - 240	0.225	0.225	0.5466	0.3961	
240 - 260	0.144	0.144	0.2268	1.7143	
220-240-260	0.252	0.126	0.7340	0.3174	3.8853

Compressive modulus

Temperatures	Sum of Squares	Mean Square	p-value	F-value	F-critical
180 - 200	1177.225	1177.225	0.1191	3.0464	5.3177
200 - 220	24970.009	24970.009	3.7872×10^{-5}	66.5779	
220 - 240	272.484	272.484	0.2811	1.3359	
240 - 260	0.1	0.1	0.9717	0.0013	
220-240-260	370.4053	185.2027	0.3313	1.2131	3.88533

Figure S1. Specimen for tensile test with the marks of the dimensional measurements.

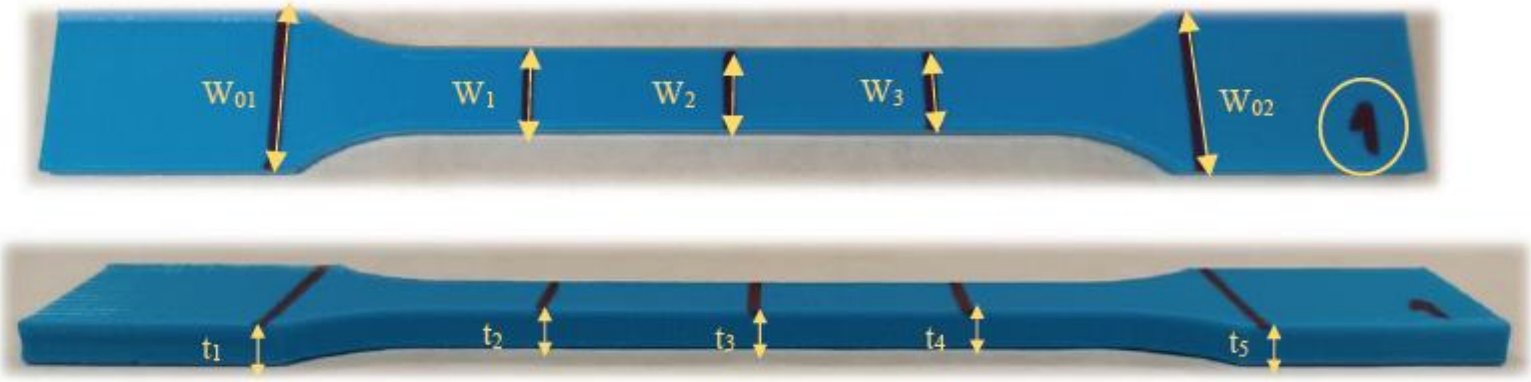

Figure S2. Specimen for flexural test with the marks of the dimensional measurements.

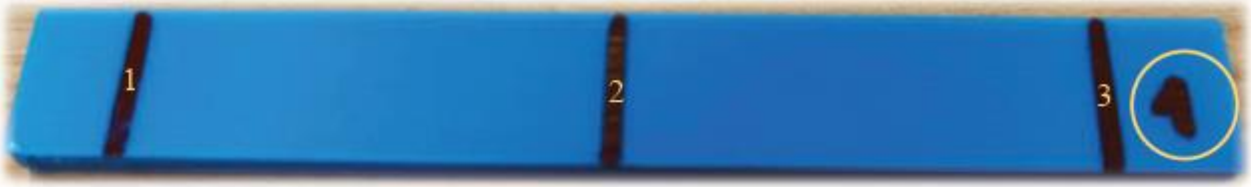

Figure S3. Specimen for compression test with the marks of the dimensional measurements.

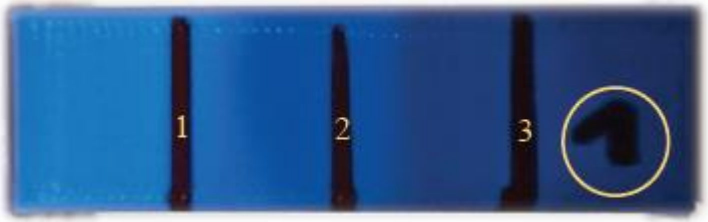

Figure S4. Optical images of the fracture surface corresponding to the tensile tested horizontal printed specimens.

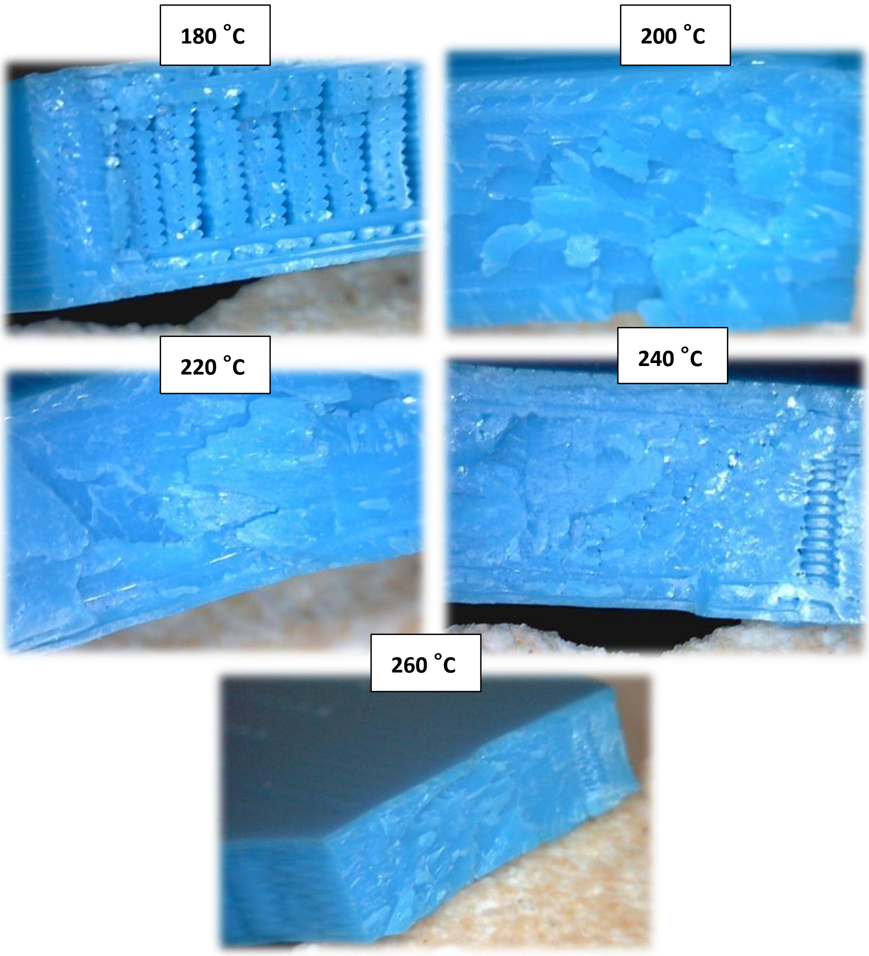

Figure S5. Optical images of the fracture surface corresponding to the tensile tested transversal printed specimens.

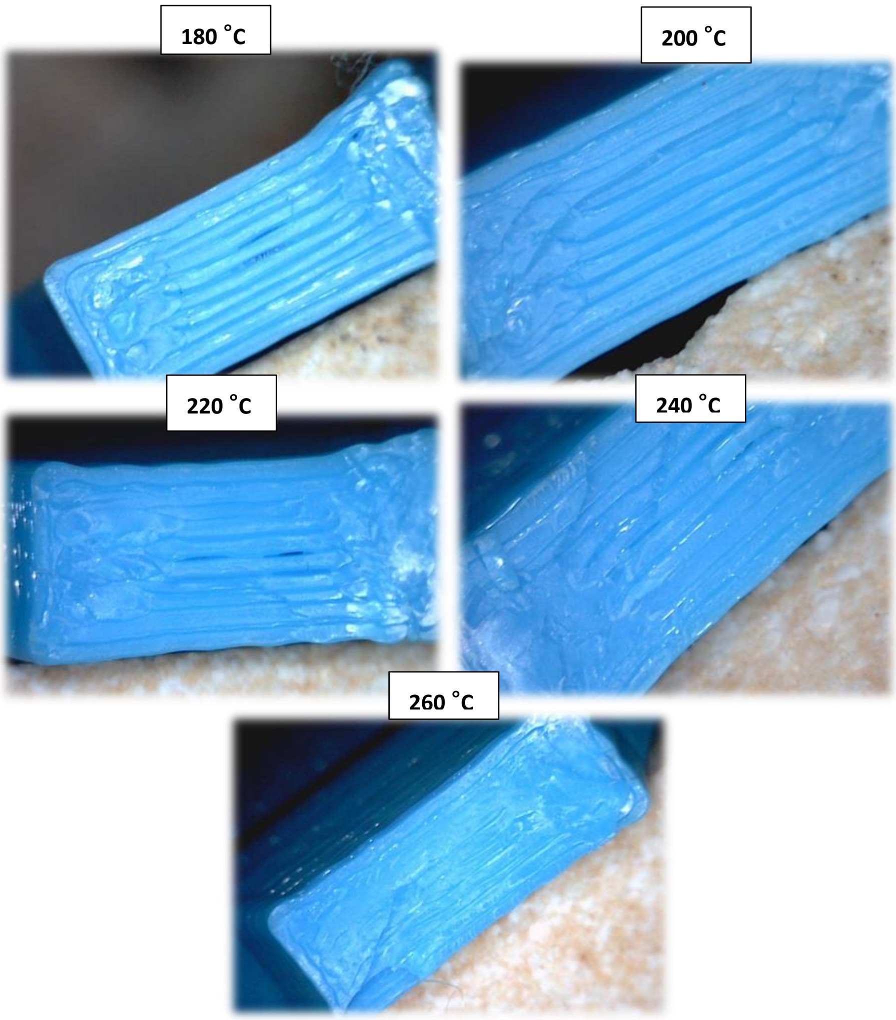

Figure S6. Optical images of the fracture surface corresponding to the flexural tested specimens.

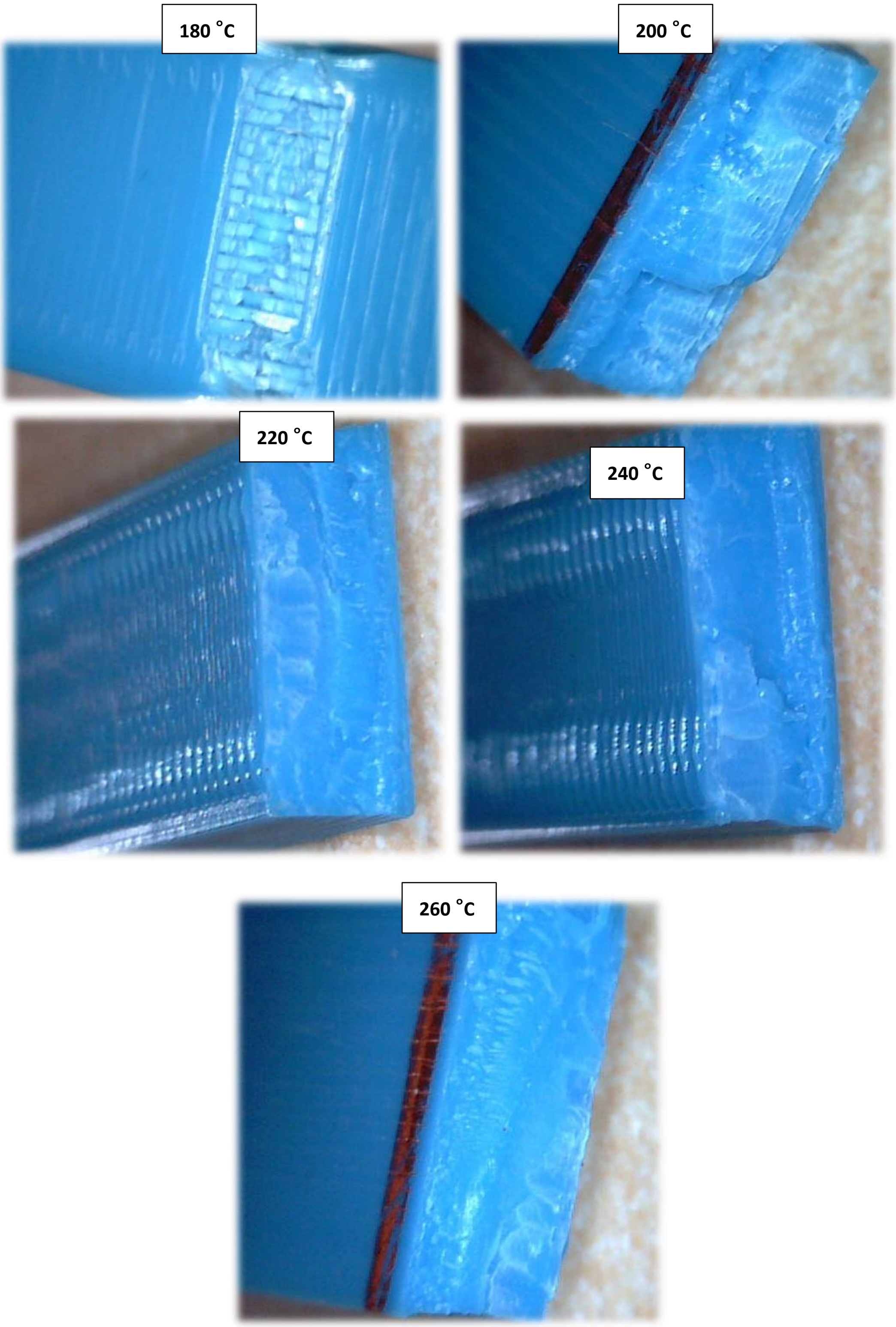

Figure S7. Representative of the complete stress-strain curves, until 45 kN of load, for the compression specimens.

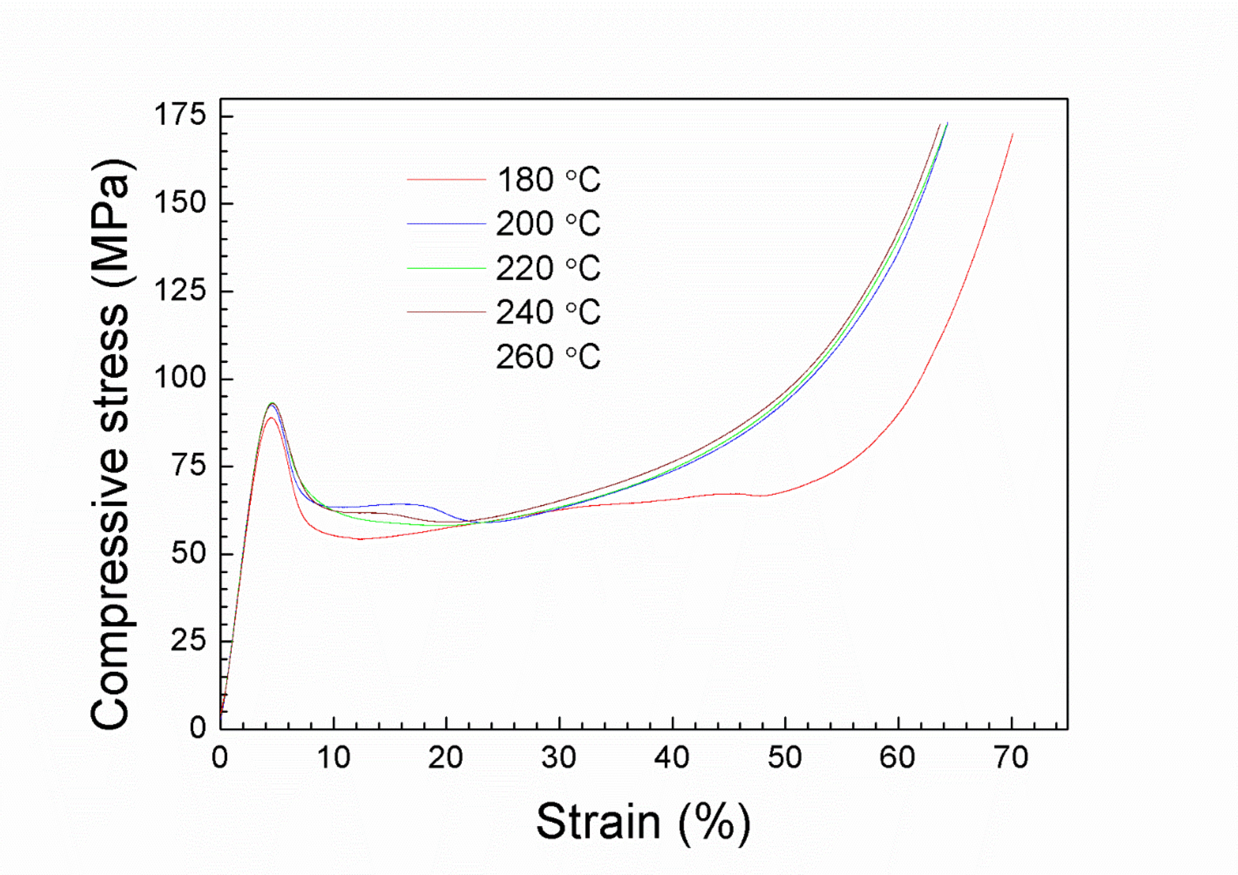

Figure S8. Optical images of the tested compression specimens.

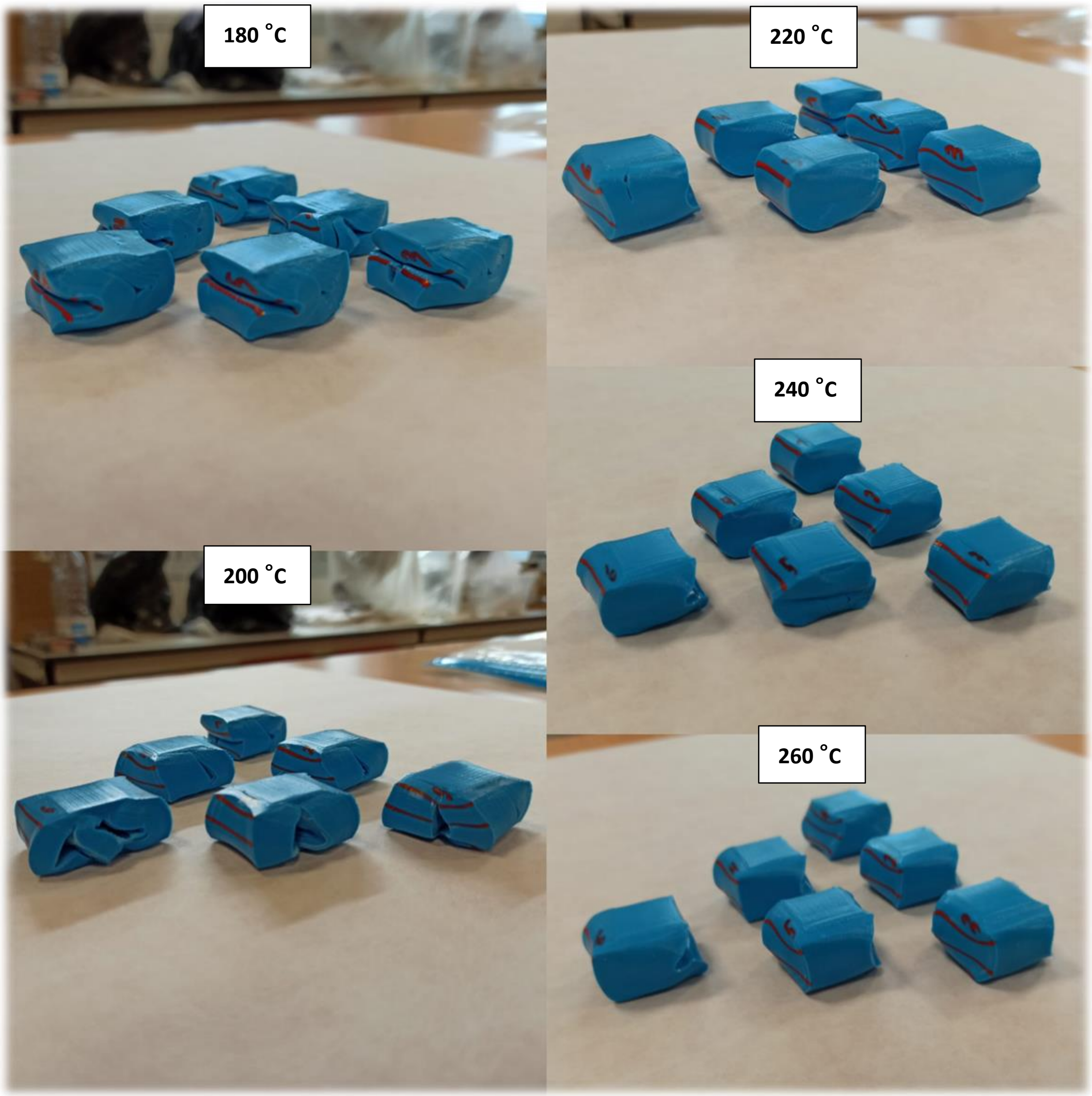

Supplement: Supplementary file 1 [file polymers-16-01867-s001.zip › polymers-3059365-supplementary.pdf]
